# Pentapeptide repeat protein QnrB1 requires ATP hydrolysis to rejuvenate poisoned gyrase complexes

**DOI:** 10.1093/nar/gkaa1266

**Published:** 2021-01-12

**Authors:** Łukasz Mazurek, Dmitry Ghilarov, Elizabeth Michalczyk, Zuzanna Pakosz, Mikhail Metelev, Wojciech Czyszczoń, Karolina Wawro, Iraj Behroz, Svetlana Dubiley, Roderich D Süssmuth, Jonathan G Heddle

**Affiliations:** Malopolska Centre of Biotechnology, Jagiellonian University, Krakow, Poland; Postgraduate School of Molecular Medicine, Warsaw, Poland; Malopolska Centre of Biotechnology, Jagiellonian University, Krakow, Poland; Malopolska Centre of Biotechnology, Jagiellonian University, Krakow, Poland; Malopolska Centre of Biotechnology, Jagiellonian University, Krakow, Poland; Postgraduate School of Molecular Medicine, Warsaw, Poland; Institute of Gene Biology, Moscow, Russia; Malopolska Centre of Biotechnology, Jagiellonian University, Krakow, Poland; Malopolska Centre of Biotechnology, Jagiellonian University, Krakow, Poland; Institute of Biological Chemistry, Technische Universität Berlin, Berlin, Germany; Institute of Gene Biology, Moscow, Russia; Institute of Biological Chemistry, Technische Universität Berlin, Berlin, Germany; Malopolska Centre of Biotechnology, Jagiellonian University, Krakow, Poland

## Abstract

DNA gyrase, a type II topoisomerase found predominantly in bacteria, is the target for a variety of ‘poisons’, namely natural product toxins (e.g. albicidin, microcin B17) and clinically important synthetic molecules (e.g. fluoroquinolones). Resistance to both groups can be mediated by pentapeptide repeat proteins (PRPs). Despite long-term studies, the mechanism of action of these protective PRPs is not known. We show that a PRP, QnrB1 provides specific protection against fluoroquinolones, which strictly requires ATP hydrolysis by gyrase. QnrB1 binds to the GyrB protein and stimulates ATPase activity of the isolated N-terminal ATPase domain of GyrB (GyrB43). We probed the QnrB1 binding site using site-specific incorporation of a photoreactive amino acid and mapped the crosslinks to the GyrB43 protein. We propose a model in which QnrB1 binding allosterically promotes dissociation of the fluoroquinolone molecule from the cleavage complex.

## Graphical Abstract

**Graphical Abstract:**
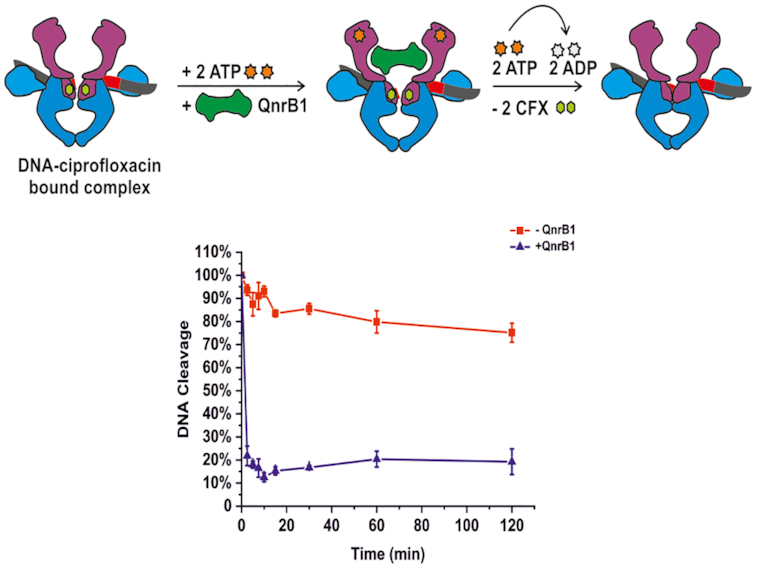
Pentapeptide repeat protein QnrB1 requires ATP hydrolysis to rejuvenate poisoned gyrase complexes.

## INTRODUCTION

**Figure 1.**
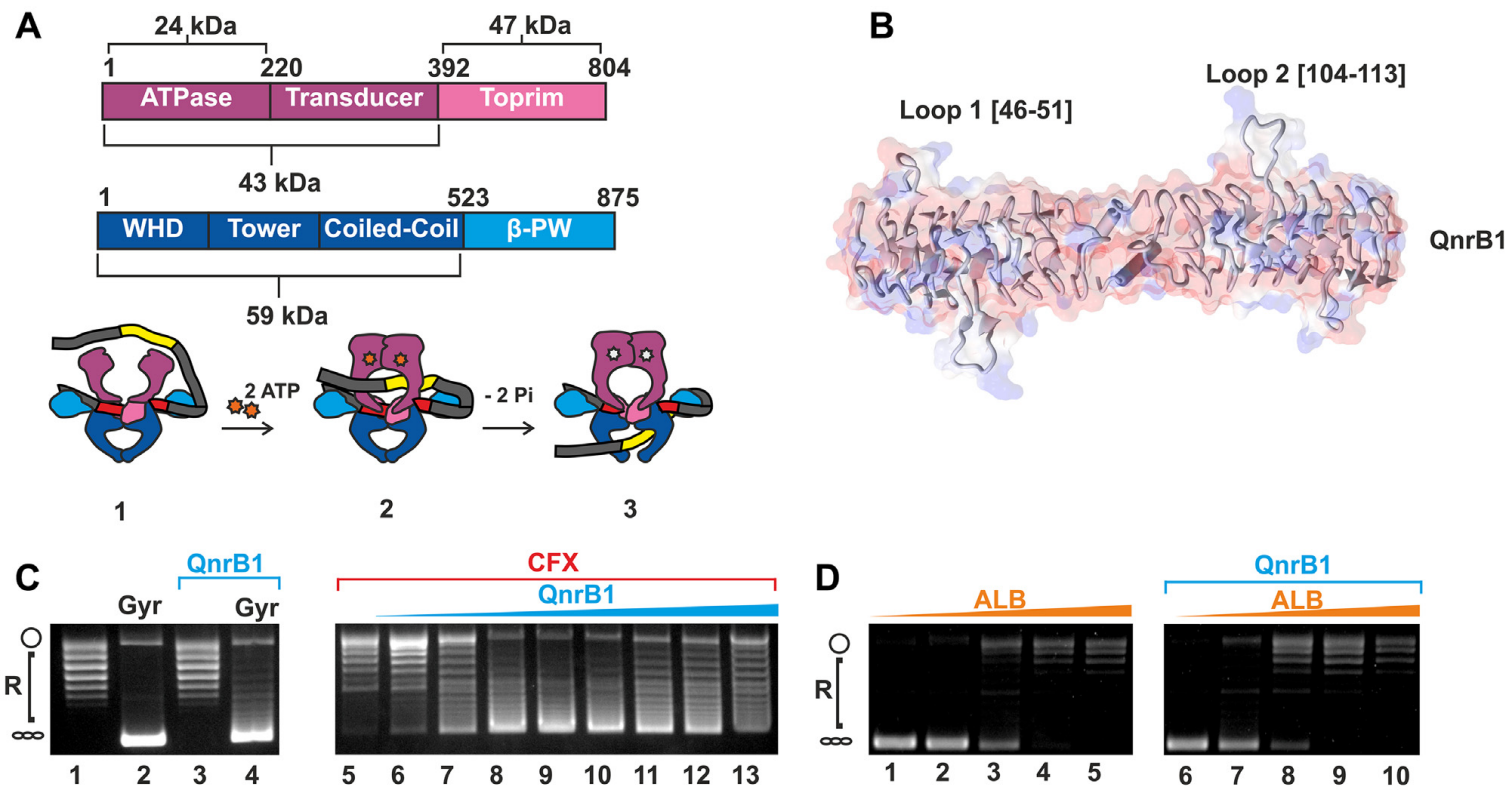
Poison-specific gyrase rescue mediated by QnrB1 protein. (**A**) *Top:* Domain architecture of *E. coli* DNA gyrase. Gyrase B is composed of ATPase, transducer (both purple) and TOPRIM (pink) domains. Indicated are molecular masses of different protein fragments (24, 43 and 47 kDa) produced and used in this work. Gyrase A is composed of winged-helix (WHD), tower, coiled-coil (all three blue) and β – pinwheel (cyan) domains. *Below*: A scheme of DNA gyrase mechanism. (**1**) A double-stranded DNA (G-segment (red)) is captured by the DNA gate, the ATPase gate is open to allow enter of the T-segment (yellow); (**2**) ATP binding leads to dimerization of the ATPase domains and trapping of the T-segment in the upper cavity; (**3**) this is followed by ATP hydrolysis, G-segment cleavage, DNA-gate opening and T-segment translocation. (**B**) Cartoon and molecular surface (transparent) representations of QnrB1 (PDB: 2XTW). Indicated are looped regions, previously found to be implicated in protective activity. (**C**). Plasmid supercoiling assay showing: lane 1: relaxed pBR322, lane 2: supercoiling by 1 U of gyrase, lane 3: lack of detectable nuclease activity in the purified QnrB1 (50 μM QnrB1) and lane 4: partial inhibition of gyrase by 50 μM QnrB1; lanes 6–13: gyrase inhibition by CFX (5 μM) and rescue by increasing concentrations of QnrB1 (0.04, 0.2, 1, 5, 10, 20, 25, 50 μM). Positions of negatively supercoiled and relaxed DNA are indicated by the graphics at the left (same notation used in other figures). (**D**). The gel shows gyrase supercoiling inhibition by increasing concentrations of albicidin in lanes 1–5 (0.016, 0.16, 1.6, 16, 160 μM). The effect of the addition of 5 μM QnrB1 to the same reactions (lanes 6–10).

DNA gyrase is an essential enzyme responsible for the maintenance of the bacterial chromosome in a negatively supercoiled state and for removal of torsion accumulated in front of DNA and RNA polymerases (1,2). Gyrase functions as a heterotetramer consisting of two GyrA and two GyrB subunits, with three interfaces (‘gates’) between them (Figure 1A). The supercoiling mechanism of gyrase has been extensively studied and is understood in some detail (1,3–4) (Figure 1A). Briefly, double-stranded DNA is wrapped around the C-terminal domains (CTD) of GyrA such that one DNA segment (the gate segment, G) is bound across the ‘DNA gate’ (formed by the N-terminal winged helix domains (WHD) of GyrA and the C-terminal topoisomerase-primase (TOPRIM) domains of GyrB) whilst a more distal segment of the same DNA molecule (T, or transported segment) forms a positive node with the G segment. In the presence of Mg^2+^, DNA is cleaved by the two active-centre Tyr residues of the GyrAs, each of which forms a new phosphodiester bond with the cleaved fragment. ATP binding to the N-terminal ATPase domains of GyrB induces their dimerization. This leads to the capture of the T-segment and its passage through the cleaved G segment and out of the bottom gate of the enzyme.

The most effective gyrase-targeting agents are the so-called ‘poisons’, which trap the enzyme in a post G-segment cleaved state. Accumulation of these stalled enzyme complexes on DNA leads to cell death (5). One example are fluoroquinolones (FQs), a particularly successful class of antibacterials, with a broad-range bactericidal activity. FQs intercalate between the DNA bases and are anchored to the Ser83 (*Escherichia coli* numbering) and Glu87 residues of GyrA via a Mg^2+^ ion (6,7). While mutations in GyrA account for the majority of target-related resistance to FQs, concerns have grown over the spread of plasmid-borne resistance mediated by *qnr* (quinolone resistance) genes (8). Although resistance conferred by *qnr* genes is incomplete, it facilitates the emergence of chromosomal mutations (9). *Qnr* family members encode pentapeptide repeat proteins (PRPs), composed of tandem 5-amino acid repeats of general sequence A(D/N)LXX (10). A homologous protein, MfpA, found in *Mycobacterium tuberculosis* increases resistance of this organism to FQs (11). Likewise, AlbG from *Xanthomonas albilineans* and McbG from *E. coli* (12,13) are thought to protect the host gyrase against specific gyrase-targeting natural products, namely the polyketide antibiotic albicidin (ALB) (14) and the post-translationally modified peptide microcin B17 (MccB17) (15–17). Similar to FQs, ALB and MccB17 stabilize the gyrase cleavage complex, but the detailed mechanism is not known. Partial cross-resistance with quinolones was also reported for both albicidin and MccB17 (14,18), suggesting that the binding sites for the compounds may overlap.

Structures of AlbG, MfpA, QnrB1 and other topoisomerase-interacting PRPs have been solved by X-ray crystallography (12,19–22). All these proteins fold into rod-like β-barrels with a square cross-section and dimerize via a C-terminal α-helix. Structure-function analysis of QnrB1 highlighted the significance of loops protruding from the helical scaffold (loops 1 and 2) as their deletion abrogated protective activity (Figure 1B) (20,22,23). Various hypotheses have been proposed to explain the effects of QnrB1 and related PRPs. The right-helical shape and stretches of negative charge along the length of MfpA and other PRPs are similar to that of double-stranded DNA, leading to the idea that PRPs can act as G-segment DNA mimics (19). In this concept, as a result of competition between the PRP and G-segments for gyrase binding, there would be fewer complexes with DNA present, hence fewer lethal double stranded DNA breaks. In agreement with this hypothesis, MfpA was shown to inhibit gyrase *in vitro* (19,22) and Qnr protein was shown to reduce DNA binding to gyrase in a filter binding assay (24). However, later it was shown that QnrB1 does not inhibit gyrase supercoiling activity, at least at the concentrations required for rescue from the effects of FQs (20–21,25). This observation is incompatible with a model in which PRPs compete with G-segment binding, as clearly supercoiling cannot proceed without G-segment present. This led to the idea of a direct recognition of the gyrase-DNA-drug complex by the PRP resulting in dissociation of the drug (20). These observations and apparent DNA-mimicry can be reconciled by the proposed ‘T-segment mimicry’ model, where the transport of the PRP through the enzyme destabilizes the enzyme-drug complex and allows for dissociation of the drug from the enzyme (26).

In order to clarify the mechanisms whereby PRPs protect gyrase from poisons, we have analyzed the protective effects of QnrB1, AlbG and McbG toward their cognate target toxins (CFX, ALB or MccB17 respectively) *in vivo* using *E. coli* DNA gyrase as a model. We have shown that the protective effect in each case is poison-specific. Further detailed experiments with purified QnrB1 protein showed that the rescue effect strictly requires ATP hydrolysis by gyrase and leads to the destabilisation of the enzyme-drug complex. Our data are not compatible with the G-segment displacement model but agree with the models where interactions of PRP with gyrase are transient and drug-specific.

## MATERIALS AND METHODS

### Bacterial strains, plasmids and molecular cloning

*Escherichia coli* DY330 GyrA-SPA (sequential peptide affinity) (W3110 Δ*lacU169 gal490 λcI857* Δ(cro-bioA) *gyrA*-SPA) and GyrB-SPA (W3110 Δ*lacU169 gal490 λcI857* Δ(cro-bioA) *gyrB*-SPA) and BW25113 were gifts from Dmitry Sutormin (Skolkovo Institute of Science and Technology) (27). All plasmids and primers used in the study can be found as Supplementary Tables S2 and S3 respectively. Plasmids pBAD-*mcbABCDEFG* (*wt* MccB17 operon) (28) and pET28-*qnrB1* (encoding QnrB1 with a C-terminal 6xHis tag) were gifts from Dr. Mikhail Metelev (Uppsala University). Plasmids pET21-GyrA and pET21-GyrB coding for full-length untagged GyrA and GyrB subunits amplified from E. coli MG1655 by PCR and cloned between Nde I and Bam HI sites were previously produced in the laboratory. Plasmids pET21-3xFLAG-GyrB and pET21-GyrA-FLAG were constructed by amplifying GyrA and GyrB genes (as above) with N-terminal (GyrB) 3xFLAG tag and C-terminal (GyrA) FLAG tags and cloning using Nde I and Xho I sites into pET21. pET28-GyrB47 was constructed by amplifying the coding sequence for the gyrase TOPRIM domain (393-804) from *E. coli* MG1655 and cloning it into pET28 using Nde I and Xho I restriction sites behind the N-terminal hexahistidine tag. pAJR10.18 (GyrA59) and pAJ1 (GyrB43) were gifts of A. Maxwell (John Innes Centre). For pBAD-*albG*, pBAD-*mcbG* and pBAD-*qnrB1*, corresponding genes were amplified from the corresponding template plasmids and cloned into pBAD vectors using Nco I and Xho I restriction sites.

### Purification of gyrase subunits and domains

*Escherichia coli* full-length GyrA and GyrB subunits were purified similarly to (29). Plasmids pET21b containing corresponding genes were transformed into BL21 (DE3) Gold (Agilent). For GyrA, 2 l of TB culture supplemented with 100 μg/ml ampicillin was incubated at 37°C with shaking to OD_600_ = 0.75, induced with 500 μM isopropyl-β-d-thiogalactopyranoside (IPTG) and incubated for a further 4 h at 37°C. Cells were harvested by centrifugation at 6000 g for 15 min at 4°C. Pellets were resuspended in buffer A (50 mM Tris–HCl pH 7.5, 10% glycerol, 1 mM EDTA, 2 mM DTT) and supplemented with protease inhibitor cocktail (Pierce). Cells were lysed using a French press and cell debris removed by centrifugation at 40 000 g for 20 min at 4°C. Clarified lysate was loaded onto 10 ml Q XL column (Cytiva) in buffer A, washed with 10 CV of buffer A, 10 CV of buffer A supplemented with 0.1 M NaCl and eluted with gradient (0.1–1 M) of NaCl in buffer A over five CVs. Collected fractions were dialyzed into buffer A overnight at 4°C and further purified by MonoQ HR 16/10 (Cytiva) using the same method as described for Q XL. Peak fractions were pooled together, concentrated using Amicon concentrators (Millipore) and loaded onto a Superdex 200 Increase 10/300 (Cytiva) column equilibrated in buffer A. Fractions containing GyrA were aliquoted, frozen in liquid nitrogen and stored at −80°C in buffer A. For GyrB, 2 l of TB culture was grown at 37°C with shaking to OD_600_ = 0.8, induced with 500 μM IPTG and incubated for a further 3 h at 28°C. Cells were lysed and processed in buffer A as described for GyrA and protein was purified using heparin affinity (Heparin FF 16/10 column, Cytiva) and anion exchange (MonoQ HR 16/10) chromatography, eluting with a 0–1 M gradient of NaCl. Peak fractions from MonoQ were pooled, dialyzed into buffer A, aliquoted and frozen at −80°C. FLAG-tagged versions of both GyrA and GyrB were purified similarly.

GyrB 43 kDa domain (GyrB43) was purified similarly to (30). The plasmid pAJ1 (31) was transformed into BL21 (DE3) Gold cells. A 1 l culture of TB inoculated with transformed cells was incubated at 37°C with shaking to OD_600_ = 0.8 after which it was induced with 500 μM IPTG. The temperature was reduced to 25°C and the culture was incubated with shaking overnight. Cells were harvested and processed similarly to GyrA and GyrB proteins. GyrB43 was first purified on a Q XL column similarly to GyrA and B proteins. Fractions containing protein of interest were combined and (NH_4_)_2_SO_4_ was added to 1.5 M. Salt-adjusted protein was loaded onto a 10 ml Phenyl Sepharose HS FF (Cytiva) column equilibrated in 1.5 M (NH_4_)_2_SO_4_ and protein was eluted by 20 CV gradient from 1.5 M to 0 (NH_4_)_2_SO_4_. Collected fractions were pooled together, dialyzed overnight in buffer A and loaded onto a 20 ml MonoQ column as described for GyrA and GyrB proteins. Peak fractions from MonoQ were pooled, dialyzed overnight into buffer A, concentrated to 10 mg/ml, aliquoted, frozen in liquid N_2_ and stored at −80°C.

GyrB47 purification was similar to (32). A 2 l culture of BL21 (DE3) Gold pET28-GyrB47 was grown in TB at 37°C. At OD_600_ = 0.75, IPTG was added to 0.5 mM and cells were grown for a further 4 h at 37°C. After harvesting, cells were resuspended in lysis buffer (50 mM Tris–Cl pH 7.5, 150 mM NaCl, 20 mM imidazole, 10% glycerol) and lysed using a French press. Cleared lysate was loaded on a pre-equilibrated 5 ml HisTrap FF column (Cytiva), washed with the lysis buffer and eluted with the lysis buffer containing 250 mM imidazole. Peak fractions were dialyzed overnight into buffer A and further purified on a 5 ml Q HP column (Cytiva). A step gradient of NaCl was used and protein eluted at 40% NaCl.

GyrA59 was purified similar to (33). Plasmid pAJR10.18 was transformed into BL21(DE3) Gold cells. Two liters of culture was grown in LB at 37°C to OD_600_ = 0.85, induced with 0.5 mM IPTG, and grown for further 4 h at 37°C. Cells were lysed by sonication in buffer A. Lysate was cleared by centrifugation at 87 000 g for 30 min at 4°C and GyrA59 was purified first on 16/10 Heparin FF (Cytiva) column using 0.1–1 M gradient of NaCl in buffer A. After overnight dialysis to buffer A, GyrA59 was further purified on a 16/10 MonoQ column using a 0–0.7M NaCl gradient over six CV. Fractions containing GyrA59 were pooled, concentrated and loaded onto a Superdex 200 16/600 gel filtration column. Pure protein, concentrated to ∼5 mg/ml was aliquoted, flash-frozen in liquid N_2_ and stored at −80°C.

### QnrB1 purification

Plasmid pET28-QnrB1 containing the *qnrB1* open reading frame with a C-terminal hexa-histidine tag was transformed into BL21 (DE3) Gold cells. 3 l of TB liquid media supplemented with 30 μg/ml kanamycin were inoculated with 30 ml of starter overnight culture and incubated at 37°C with shaking to OD _600_ = 0.8. After induction with 500 μM IPTG the temperature was reduced to 24°C and the cultures were incubated overnight with shaking. Cells were harvested by centrifugation at 7000 g for 30 min at 4°C. Pellets were resuspended in QnrB1 lysis buffer (50 mM Tris–Cl pH 8.0, 200 mM (NH_4_)_2_SO_4_, 10% glycerol, 20 mM imidazole) supplemented with protease inhibitors (Pierce). Resuspended cells were incubated for 30 min on ice with 1 mg/ml lysozyme with periodic mixing. Cells were lysed using a French press and lysate was cleared by centrifugation at 87 000 g for 30 min at 4°C. QnrB1 was purified by Ni-affinity chromatography (HisTrap HP 5 ml, Cytiva). The column was equilibrated with QnrB1 lysis buffer and after loading the lysate washed with 20 CVs of wash buffer (50 mM Tris–Cl pH 8.0, 200 mM (NH_4_)_2_SO_4_, 10% glycerol, 50 mM imidazole). The fractions were eluted with an elution buffer (50 mM Tris–Cl pH 8.0, 200 mM (NH_4_)_2_SO_4_, 10% glycerol, 250 mM imidazole). Fractions containing protein were pooled and dialyzed overnight against buffer A-Arg (50 mM Tris–HCl pH 7.5, 50 mM arginine hydrochloride, 10% glycerol, 1 mM EDTA, 2 mM DTT) and loaded on a MonoQ HR 16/10 ion exchange column (Cytiva). Peak fractions eluted over a 0–1 M NaCl gradient were pooled, concentrated, and loaded onto a 10/300 Superdex S75 Increase column (Cytiva) previously equilibrated with QnrB1 storage buffer (20 mM Tris pH 7.5, 50 mM NaCl, 5% glycerol, 50 mM arginine hydrochloride, 2 mM DTT). Peak fractions were concentrated, flash-frozen in liquid N_2_ and stored at −80°C.

### Purification of QnrB1 *para*-benzoyl-phenylalanine (*p*Bpa) mutants

Plasmids pBAD-*qnrB1[x]pBpa*, carrying *qnrB1* amber mutants with N-terminal 6xHis, were transformed into One Shot BL21 Star (DE3) cells (ThermoFisher) together with the pEVOL-*p*Bpa plasmid (34). 100 ml of LB liquid media supplemented with 30 μg/ml kanamycin and 100 μg/ml ampicillin was inoculated using a 1:100 ratio of starter overnight culture and incubated at 37°C with shaking to OD_600_ = 0.3. At this point, *para*-benzoyl-phenylalanine (*p*Bpa) was added to a final concentration of 1 mM. The incubation was continued until the culture reached OD_600_ = 0.6. The expression of orthogonal aaRS and QnrB1 was induced by the addition of arabinose (10 mM). After 6 h of expression at 37°C with shaking, the cells were collected by centrifugation at 7000 g for 30 min at 4°C. Pellets were resuspended in QnrB1 lysis buffer (50 mM Tris–Cl pH 8.0, 200 mM (NH_4_)_2_SO_4_, 10% glycerol, 20 mM imidazole) supplemented with protease inhibitors (Pierce). Resuspended cells were incubated with mixing for 30 min on ice with 1 mg/ml lysozyme. Cells were lysed by sonication and lysate was cleared by centrifugation at 87 000 g for 30 min at 4°C. Lysate was loaded onto a Ni^2+^-affinity chromatography column (5 ml HisTrap HP, Cytiva) equilibrated with QnrB1 lysis buffer. After loading the lysate, the column was washed with 20 column volumes of wash buffer (50 mM Tris–Cl pH 8.0, 200 mM (NH_4_)_2_SO_4_, 10% glycerol, 50 mM imidazole). The fractions were eluted with the elution buffer (50 mM Tris–Cl pH 8.0, 200 mM (NH_4_)_2_SO_4_, 10% glycerol, 250 mM imidazole). Fractions containing protein were subsequently diluted with the storage buffer (20 mM Tris–Cl pH 7.5, 50 mM NaCl, 5% glycerol, 50 mM arginine hydrochloride), concentrated to desired concentration and stored at −80°C.

### Purification of gyrase-targeting toxins

Microcin B17 was obtained from *E. coli* BW25113 transformed with *pBAD-mcbABCDEFG* plasmid using a method described previously (28). Briefly, transformants were cultured in 2 l of 2× YT media until OD_600_ = 0.7, induced with 10 mM arabinose and left overnight at 37°C. Cells were collected by centrifugation at 4000 g, resuspended in 100 mM acetic acid/1 mM EDTA solution and lysed by boiling for 10 min on a water bath. Lysate was clarified by centrifugation (12 000 g) and loaded onto an Agilent BondElute 10 g C18 cartridge, equilibrated with 0.1% TFA. The cartridge was extensively washed first with 20 CV 0.1% TFA, then with 20 CV of 10% MeCN in 0.1% TFA before elution with 30% MeCN/0.1% TFA. Eluate was dried *in vacuo*, reconstituted in 10% DMSO and further purified by HPLC using a COSMOSIL 5C18-MS-II 120 Å 5 μm, 10.0 × 150 mm column and a 10–30% gradient of MeCN. Albicidin was produced by total synthesis (35).

### MIC measurements

Minimal inhibitory concentrations of compounds were measured by broth microdilution in 96-well plates as described by the Clinical & Laboratory Standards Institute (36). All measurements were performed as triplicates. The error is expressed by standard deviation of the mean.

### Gyrase activity assays

For supercoiling assays, 1 unit of *E. coli* gyrase (1 unit defined as the amount of gyrase required for full conversion of 500 ng relaxed pBR322 DNA into the completely supercoiled form in 30 min at 37°C in a 30 μl reaction, corresponded to 4 nM concentration) was incubated in a total volume of 30 μl in assay buffer (35 mM Tris–Cl pH 7.5, 24 mM KCl, 4 mM MgCl_2_, 2 mM DTT, 1.8 mM spermidine, 1 mM ATP, 6.5% (w/v) glycerol, 0.1 mg/ml albumin) and 500 ng relaxed pBR322 DNA (Inspiralis Ltd.). where necessary enzymes or inhibitors were replaced with an equal volume of appropriate buffer. Reactions were stopped by the addition of chloroform:isoamyl alcohol (24:1) and STEB (20% sucrose, 50 mM Tris–Cl pH 8, 5 mM EDTA, 0.25 mg/ml bromophenol blue). The aqueous layers from the assays were run on 1% agarose TAE gels at 80 V for 2.5 h in 1× TAE buffer. Once complete, the gels were stained with 10 μg/ml ethidium bromide solution (Sigma) for 15 min and de-stained with 1× TAE buffer for 15 min and visualized using a gel documentation system (UVP). Quantification of the amount of supercoiled DNA was performed using Fiji software (37). Percentage of supercoiled DNA was plotted against QnrB1 concentration and the data was fitted to the equation: $\% SC = a{e^{b[ {QnrB1} ]}}$, where *a* and *b* are function parameters found after fitting the function using OriginLab Origin (Pro), Version 2020 b. IC_50_ (concentration of QnrB1 required for 50% supercoiling inhibition) was calculated from the fitted function. Gyrase relaxation assays were carried out in a similar manner, but ATP and spermidine were omitted and ∼5 U of gyrase were used. For gyrase cleavage assays ∼5 U of gyrase were used and reactions were terminated by the addition of 0.2% sodium dodecyl sulphate (SDS) and 0.2 mg/ml proteinase K, followed by incubation for 30 min at 37°C prior to chloroform extraction and gel analysis. For cleavage of short DNA fragments, 20 nM fragment (with the exception of the 76 bp fragment where 30 nM was used as indicated in figure) was incubated with 5 U of gyrase for 30 min, followed by reaction termination as described above. Reaction products were separated on 4–20% TBE polyacrylamide gels (ThermoFisher). DNA fragments (300–100 bp) were obtained by amplification from the pBR322 template, following by purification (GeneJet Gel Extraction and DNA Cleanup Micro kit, ThermoFisher). The 76 bp fragment was ordered as a pair of complementary oligos and annealed in a PCR machine.

Time courses of gyrase-mediated DNA cleavage were performed as follows: *E. coli* gyrase (100 units) was incubated at 25°C in 400 μl reactions with assay buffer (35 mM Tris–Cl pH 7.5, 24 mM KCl, 4 mM MgCl_2_, 2 mM DTT, 1.8 mM spermidine, 6.5% (w/v) glycerol, 0.1 mg/ml albumin) and 500 ng relaxed pBR322 DNA (Inspiralis Ltd.). For Ca^2+^ induced cleavage, MgCl_2_ was replaced with CaCl_2_. The buffer was supplemented with 1 mM ATP or 0.5 mM ADPNP as required. When tested compounds and/or proteins were absent in control reactions, they were replaced with an equal volume of DMSO or appropriate buffers. At selected time points, 20 μl aliquots were withdrawn and stopped by addition of 2 μl 5% SDS and 2 μl 250 mM EDTA. After the time course, collected samples were treated with proteinase K (0.2 mg/ml) for 30 min at 37°C and extracted by chloroform:isoamyl alcohol (24:1). The aqueous layers were mixed with STEB and run on 1% agarose TAE gels at 80 V for 2.5 h in TAE buffer. Once complete, the gels were stained with 10 μg/ml ethidium bromide solution (Sigma) for 15 min and de-stained with TAE buffer for 15 min and visualized using a gel documentation system (UVP).

Cleavage complex stability assays were performed as follows: an initial 60 μl reaction was set up using 80 units of gyrase in the assay buffer, 50 nM of relaxed DNA and 20 μM CFX. which was incubated at 37°C for 10 min. Then 20 ul aliquots were withdrawn and diluted 20-fold with buffer supplemented with 5 μM QnrB1and 0.5 mM ATP as required. At chosen time points 20 μl from each reaction was pipetted to a separate tube and stopped by adding 2 μl of 5% SDS and 2 μl 250 mM EDTA. After the time course, samples were treated with proteinase K (0.2 mg/ml) for 30 min at 37°C, then a chloroform–isoamyl alcohol mixture (24:1) and STEB (20% sucrose, 50 mM Tris–Cl pH 8, 5 mM EDTA, 0.25 mg/ml bromophenol blue) were added. The aqueous layers were run on 1% agarose TAE gels at 80 V for 2.5 h in TAE buffer. Once complete, the gels were stained with 10 μg/ml ethidium bromide solution (Sigma) for 15 min and de-stained with TAE buffer for 15 min and visualized using a gel documentation system (UVP).

### Electrophoretic mobility shift assays (EMSA)

147 bp pBR322 dsDNA fragment with known strong gyrase binding site (38) was produced by PCR and purified with ThermoFisher Scientific GeneJet Gel Extraction and DNA Cleanup Micro kit. 20 nM of the fragment was mixed with 0.2 μM of reconstituted gyrase in EMSA buffer (30 mM Tris–Cl pH 7.5, 75 mM KCl, 6% glycerol, 2 mM MgCl_2_, 1 mM DTT). QnrB1 were added as indicated. In control reactions, QnrB1 were replaced by an equal volume of their storage buffers. Reactions were incubated for 30 min at 25°C and run on 6% polyacrylamide gels in TBM buffer (90 mM Tris-borate, pH 7.5, 4 mM MgCl_2_) at 150V at room temperature. After the run gels were stained with SYBR Gold (ThermoFisher) for 20 min and visualized under UV light.

### ATPase assays

Gyrase and GyrB43 ATPase assays were carried out using Inspiralis kits according to the protocol provided by Inspiralis Ltd based on Tamura & Gellert (39). Each reaction contained 50 mM Tris–HCl (pH 7.5), 1 mM EDTA, 5 mM magnesium chloride, 5 mM DTT, 10% (w/v) glycerol, 0.8 mM PEP, 0.4 mM NADH and ∼1 U of PK/LDH mix (Sigma). For GyrB43 assays, the concentration of GyrB43 was 4 μM. For gyrase assays, 50 nM gyrase tetramer (A_2_B_2_) was used. Linear pBR322DNA (Inspiralis) was used at 10.5 nM where indicated. Assays were performed in microtiter plates with a reaction volume of 100 μl. The absorbance at 340 nm was measured continuously in a plate reader (SpectraMAX190, Molecular Devices) and used to evaluate the oxidation of NADH (using an extinction coefficient of 6.22 mM^−1^ cm^−1^), which relates stoichiometrically to the production of ADP. The results were described by a Michaelis – Menten equation $V = \frac{{{V_{max}}[ S ]}}{{{K_m} + [ S ]}}$ using Origin (Pro), Version 2020 b (OriginLab Corporation). Novobiocin (50 μM) was used as a control for gyrase-independent ATPase activity, which was found negligible.

### Fluorescence anisotropy measurements

QnrB1 was N-terminally labeled with AlexaFluor 488-carboxylic acid-2,3,5,6-tetrafluorophenyl ester-5 isomer (5-TFP, ThermoFisher), according to a procedure described for YacG protein (32). Briefly, purified QnrB1-His was exchanged to amino labeling buffer (25 mM HEPES–Cl pH 7.5, 10% (v/v) glycerol, 200 mM KCl and 1 mM TCEP, final pH of solution was 7.0). A 15 times molar excess of AlexaFluor 488-5-TFP over QnrB1 was added to the QnrB1 solution. The reaction was incubated in 25°C for 1 h with shaking. Unreacted dye was quenched by adding 1 M l-lysine dissolved in 20 mM Tris–HCl pH 7.5 (final lysine concentration 130 mM) and incubating the reaction at 37°C for 30 min. Free dye was separated from labeled protein using a Superdex S75 increase 10/300 GL column (Cytiva), equilibrated with QnrB1 storage buffer.

Fluorescence measurements were carried out with a RF-6000 spectrofluorimeter (Shimadzu) fitted with a polarizer. The assays were carried out at specific excitation/emission wavelengths of 488/520 nm for Alexa-488 or 600/666 nm for Cy5 using a 10 nm bandwidth. For QnrB1 binding to gyrase, 50 nM labeled QnrB1 was mixed in EMSA buffer with appropriate gyrase subunit(s) and incubated for 10 min at room temperature in darkness. When 1 mM ADPNP was used, the samples were incubated for additional 1 h at room temperature with addition of 2 mM MgCl_2_. Curves were fitted using OriginLab Origin (Pro)Version 2020b according to a standard one site specific binding equation: $Binding = \ \frac{{{B_{max}}*x}}{{Kd*x}}$.

QnrB1/DNA binding competition assays were performed as follows: 90 bp Cy5-labeled oligonucleotide encompassing the strong gyrase binding site from plasmid pBR322 was ordered from Sigma and annealed with an antisense unlabeled oligo by heating to 99°C and gradual cooling. To measure the ability of QnrB1 to compete with DNA, 20 nM 90 bp Cy5-DNA fragment complex was mixed in buffer (50 mM Tris–HCl, pH 7.5, 0.5 mM TCEP, 10 mM MgCl_2_, 30 mM KGlu, 5% glycerol) with 1 μM gyrase (A_2_B_2_) and increasing concentrations of label-free QnrB1. Optimal concentration of gyrase complex for binding experiments was established beforehand by mixing 20 nM 90 bp Cy5-DNA and increasing concentrations of gyrase in the above-mentioned buffer. To measure the ability of DNA to compete with QnrB1, 50 nM Alexa-488-labeled QnrB1 was mixed with 1 μM gyrase and an increasing concentration of label free linearized pBR322 (Inspiralis).

### Pull-down experiments

5 μM purified FLAG-QnrB1 was mixed with 0.65 μM gyrase A_2_B_2_ complex in 25 mM Tris–Cl pH 7.5, 5 mM MgCl_2_, 50 mM KCl and 5% glycerol in 20 μl volume. For the experiments with ADPNP, the nucleotide (1 mM) was added 30 min before QnrB1 to induce GyrB dimerisation and the reaction was pre-incubated at RT together with a control reaction. An equal amount of equilibrated FLAG M2 agarose (Sigma) was added to each reaction. After addition of QnrB1, reactions were incubated for a further 60 min at RT with gentle shaking. Resin was washed three times with TGE (20 mM Tris–Cl pH 7.5, 10% glycerol, 100 mM NaCl) and eluted with 50 μg/ml (final concentration) of 3× FLAG peptide (Sigma) in TGE. Load and eluate material were analyzed by SDS-PAGE.

### *In vivo* benzoyl-phenylalanine (*p*Bpa) -driven UV crosslinking

*Escherichia coli* GyrA-SPA and GyrB-SPA were co-transformed with pEVOL-*p*Bpa plasmid and pBAD-*QnrB1[x]pBpa* plasmid containing appropriate amber codon substitutions. Cells were grown at 37°C with shaking in LB medium supplemented with antibiotics and 1 mM *p*Bpa. Protein expression was induced by addition of 10 mM arabinose at OD_600_ = 0.6 and culture was allowed to grow for a further 3 h. After that time cells were centrifuged (5000 g, 10 min), the medium was removed, and cells were resuspended in phosphate buffered saline (PBS, pH 7.5). Cell suspensions were poured into plastic Petri dishes and irradiated with UV for 30 min at λ = 365 nm (8 W). The temperature of the cell suspension did not exceed 37°C during UV exposure. The samples collected before and after UV-crosslinking were subsequently analyzed by SDS-PAGE in 4–20% gradient gels (Bio-Rad TGX). Gels were subsequently transferred onto a PVDF membrane and FLAG epitopes were detected by Sigma M2 antibodies followed by ECL imaging.

### *In vitro* crosslinking experiments

400 nM (final concentration) of relevant gyrase subunits or domains (GyrB/A/B43/B47/B24) and 5 μM (final concentration) of QnrB1 *p*Bpa were combined together in EMSA buffer (75 mM KCl, 30 mM Tris–Cl pH 8, 2 mM MgCl_2,_ 6% glycerol, 1 mM DTT). After 30 min of incubation at room temperature the mixture was irradiated with UV light (λ = 365 nm) in UV-transparent Eppendorf tubes for 30 min. The temperature of the reaction was monitored and did not exceed 37°C. For time-course experiments, aliquots were taken at the indicated time points. For ADPNP containing experiments, 1 mM ADPNP was added 30 min prior to crosslinking to induce GyrB or GyrB43 dimerization. The reaction products were analyzed on SDS-PAGE gels. Competition crosslinking experiments with WT QnrB1 and QnrB1 Y123pBpa were performed as follows: 5 μM (final concentration) of QnrB1 *p*Bpa and 400 nM (final concentration) A_2_B_2_ gyrase complex and an appropriate amount of WT QnrB1 in EMSA buffer were incubated for 30 min at RT. The samples were irradiated with UV light (λ = 365 nm) in UV-transparent Eppendorf tubes for 30 min. The reaction products were analyzed on SDS-PAGE gels.

## RESULTS

### Pentapeptide repeat proteins offer specific protection against their cognate toxins *in vivo*

To compare protective activities of different PRPs in identical conditions, we created a set of arabinose-inducible pBAD plasmids, carrying coding sequences for tagless QnrB1, McbG or AlbG. *E. coli* BW25113 (*ara*-) were transformed with one of these plasmids or with an empty pBAD vector, and MICs of CFX, ALB and MccB17 were measured upon induction. Similarly to the previously published data (23), induction of QnrB1 led to a 16-fold increase of CFX MIC compared to the reference (Table 1). Induction of McbG led to a modest 4-fold increase, and no increase in CFX MIC was observed upon AlbG induction. In stark contrast, tests with ALB showed that upon AlbG induction, its MIC increased >128-fold (higher concentrations could not be tested due to the limited solubility of albicidin). Expression of QnrB1 did not increase the albicidin MIC, and expression of McbG resulted in a 4-fold increase of MIC. Finally, the MICs for MccB17 were increased 37-fold for the McbG producing strain, whereas AlbG and QnrB1 induction resulted in 6-fold and 3-fold increase of MIC, respectively. These results clearly indicated that all three PRPs provide specific protection against their cognate toxins.

**Table 1.** Measured MIC values. Measured MICs of albicidin (ALB), ciprofloxacin (CFX) and microcin B17 (MccB17) for *E. coli* BW25113 strain transformed with empty vector (pBAD) or plasmids expressing different PRPs

	Toxin					
	ALB		MccB17		CFX	
Plasmid	Obtained MIC [μM]	Fold change	Obtained MIC [μM]	Fold change	Obtained MIC [μM]	Fold change
pBAD	0.028 ± 0.005	1	0.58 ± 0.09	1	0.016 ± 0.004	1
pBAD-QnrB1	0.023 ± 0.008	1	1.3 ± 0.1	2	0.25 ± 0.06	16
pBAD-McbG	0.13 ± 0.02	5	>15	>26	0.12 ± 0.03	8
pBAD-AlbG	>10	>341	2.6 ± 0.2	4	0.016 ± 0.004	1
pBAD-AlbG Δ_91-97_	0.062 ± 0.009	2	-	-	-	-

It was previously shown that the deletion of three amino acids (107–109) from QnrB1 loop 2 compromises protection against FQs (20,23). AlbG contains a similarly located loop (91–97), and the structure of loop deletion Δ_91–97_ AlbG variant has been reported (12), but not tested for protection from albicidin. This prompted us to produce this mutant and evaluate its activity. Expression of Δ_91–97_*albG* led to a 170-fold lower MIC value for ALB, compared to the WT *albG* gene when tested *in vivo* in our system. (Table 1).

### QnrB1 rescues *E. coli* gyrase by reducing cleavage complex formation

To investigate the biochemical basis of protection offered by PRPs, we purified hexahistidine-tagged QnrB1 and first tested its activity in gyrase supercoiling assays. Similar to previous reports (20), QnrB1 provided limited protection (supercoiling was not completely restored) against 5 μM CFX (Figure 1C) with calculated EC50_QnrB1_ (concentration of QnrB1 required to observe half of maximum protective effect) 0.2 μM. Complete rescue was observed when CFX concentration was lowered to 1 μM. As can be seen from the same figure, high concentrations of QnrB1 (>10 μM) were found to inhibit gyrase activity, and this inhibitory effect was also observed without CFX present (Supplementary Figure S1A). Calculated IC_50 QnrB1_ was 11 μM, >50 times higher than EC50. The above-mentioned Δ_107–109_ mutant of QnrB1 (QnrB1 ΔTTR) showed the same level of supercoiling inhibition as WT QnrB1 but was unable to rescue gyrase supercoiling inhibited by CFX (Supplementary Figure S1BC). Akin to the *in vivo* results, we saw no protection when we swapped the inhibitors and tested protection by QnrB1 against ALB (Figure 1D).

**Figure 2.**
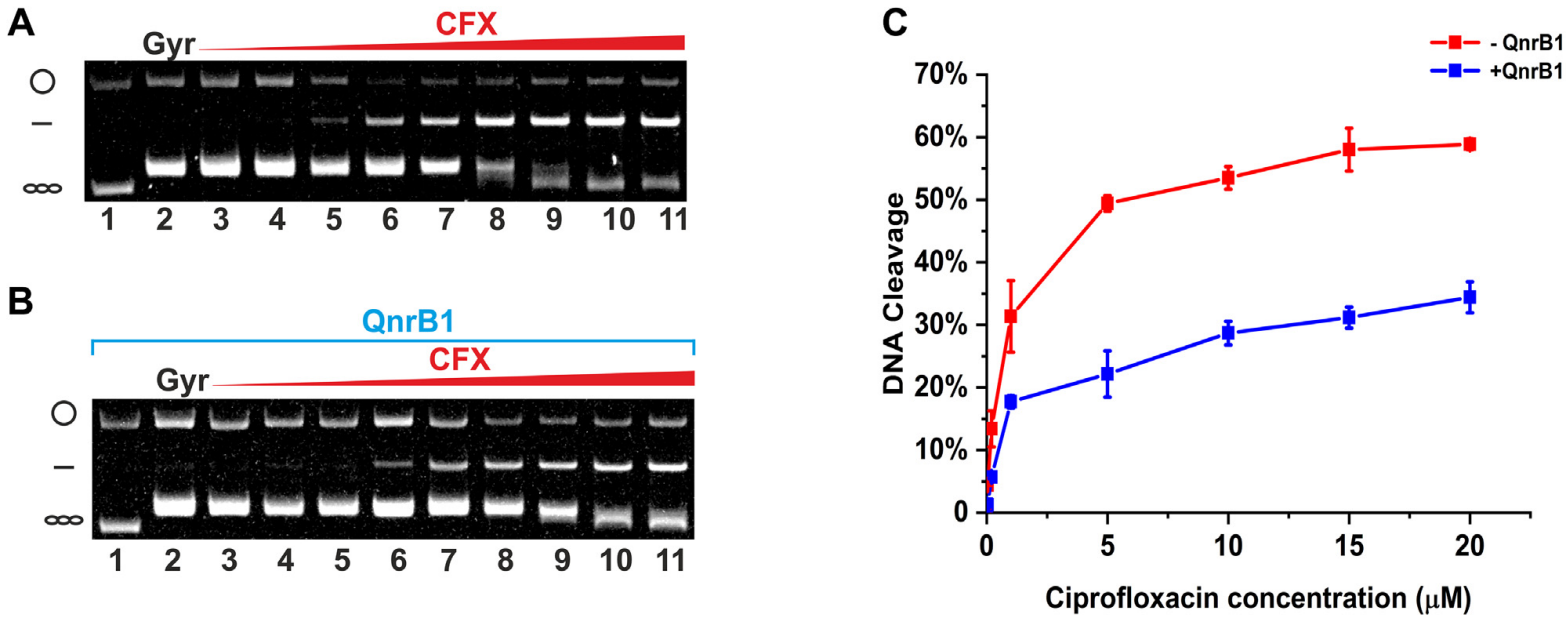
QnrB1 decreases the amount of cleaved DNA. Gyrase cleavage assay shows that QnrB1 offers protection from cleavage induced by ciprofloxacin. (**A**) DNA gyrase (5 U) cleavage reactions with increasing amounts of ciprofloxacin run in the presence of ethidium bromide (EtBr). (**B**) DNA gyrase (5 U) cleavage reactions with increasing amounts of ciprofloxacin in presence of 5 μM QnrB1, run on an EtBr gel. Lane 1, relaxed pBR322. Lane 2, relaxed pBR322 with DNA gyrase. Lanes 3–11, relaxed pBR322 gyrase and increasing concentrations of ciprofloxacin (0.0016, 0.0008, 0.04, 0.2, 1, 5, 10, 15, 20 μM) (**C**) Cleaved DNA in the absence (*blue*) and presence (*red*) of QnrB1 quantified and plotted. Error bars represent standard deviation (SD) of 3 independent experiments.

It was previously shown that QnrB1 reduced the amount of DNA gyrase complexes with cleaved DNA (DNA cleavage) stabilized by CFX (20). We have confirmed this result, showing that in the cleavage assay, the amount of linear DNA in the presence of 5 μM QnrB1 is decreased ∼50% across concentrations of ciprofloxacin tested (up to 20 μM) (Figure 2A and C). Inhibition of FQ-induced cleavage was also observed when negatively supercoiled DNA was used as a substrate, but the effect was weaker (Supplementary Figure S2AB).

As GyrB ATPase domains and GyrA CTDs are dispensable for FQ-stimulated cleavage, we produced the previously reported truncated gyrase complexes: GyrA59_2_/B_2_ and GyrA_2_/B47_2_ and tested if DNA cleavage by these complexes (using negatively supercoiled DNA as a substrate) can be inhibited by QnrB1. GyrA59_2_/B_2_ complex which lacks the GyrA CTDs can relax negatively supercoiled DNA using ATP, similarly to Topo IV (40). In contrast, GyrA_2_/B47_2_ which lacks the GyrB43 ATPase-transducer domain, is capable of ATP-independent relaxation (41). The protective effect of QnrB1 was seen only in cleavage assays with A59_2_/B_2_ (Supplementary Figure 2CD), where the amount of linear DNA trapped by CFX was observed to decrease in the presence of 5 μM QnrB1. No effect was observed for A_2_/B47_2_ (not shown). We concluded that DNA wrapping is not required for the QnrB1 to act, but the ATPase domain is indispensable. To further investigate the potential role of DNA wrapping, we tested short linear DNA fragments (76, 100, 133, 147, 220 or 300 bp) as a substrate for full-length gyrase cleavage (Supplementary Figure S3); again, reduction of cleavage was clearly visible even with the shortest fragment tested, meaning that QnrB1 indeed does not require a DNA node to act.

### QnrB1 decreases DNA binding to gyrase

The observed specific protective action of QnrB1 seems inconsistent with the G-segment DNA mimicry model, where PRPs compete with DNA for binding to the enzyme; however, such DNA mimicry could explain the weak inhibitory effect of QnrB1. We carried out EMSA experiments to test whether QnrB1 could influence DNA binding by gyrase. In these assays, QnrB1 decreased the affinity of gyrase to dsDNA (147 bp fragment, encompassing the strong gyrase site from plasmid pBR322) with an IC_50_ (concentration required to decrease the amount of bound DNA by half) of 10.93 ± 0.58 μM, which is very close to the IC_50_ of 11.25 μM observed in the supercoiling assays (Supplementary Figure S4AB). In the presence of CFX, which stabilizes DNA binding, QnrB1 could not outcompete DNA (Supplementary Figure S4C). Additional fluorescence anisotropy experiments using either labeled QnrB1 or labeled 90 bp pBR322 DNA fragment also showed competition between linear DNA and QnrB1 (Supplementary Figure S4DE), occurring at a similar concentration range. Therefore, DNA mimicry cannot explain the ability of QnrB1 to rescue gyrase supercoiling and inhibit DNA cleavage, which is manifested at much lower QnrB1 concentrations. However, it can account for the high concentration inhibitory effect of QnrB1.

### QnrB1 requires ATP hydrolysis to rejuvenate poisoned gyrase complexes

**Figure 3.**
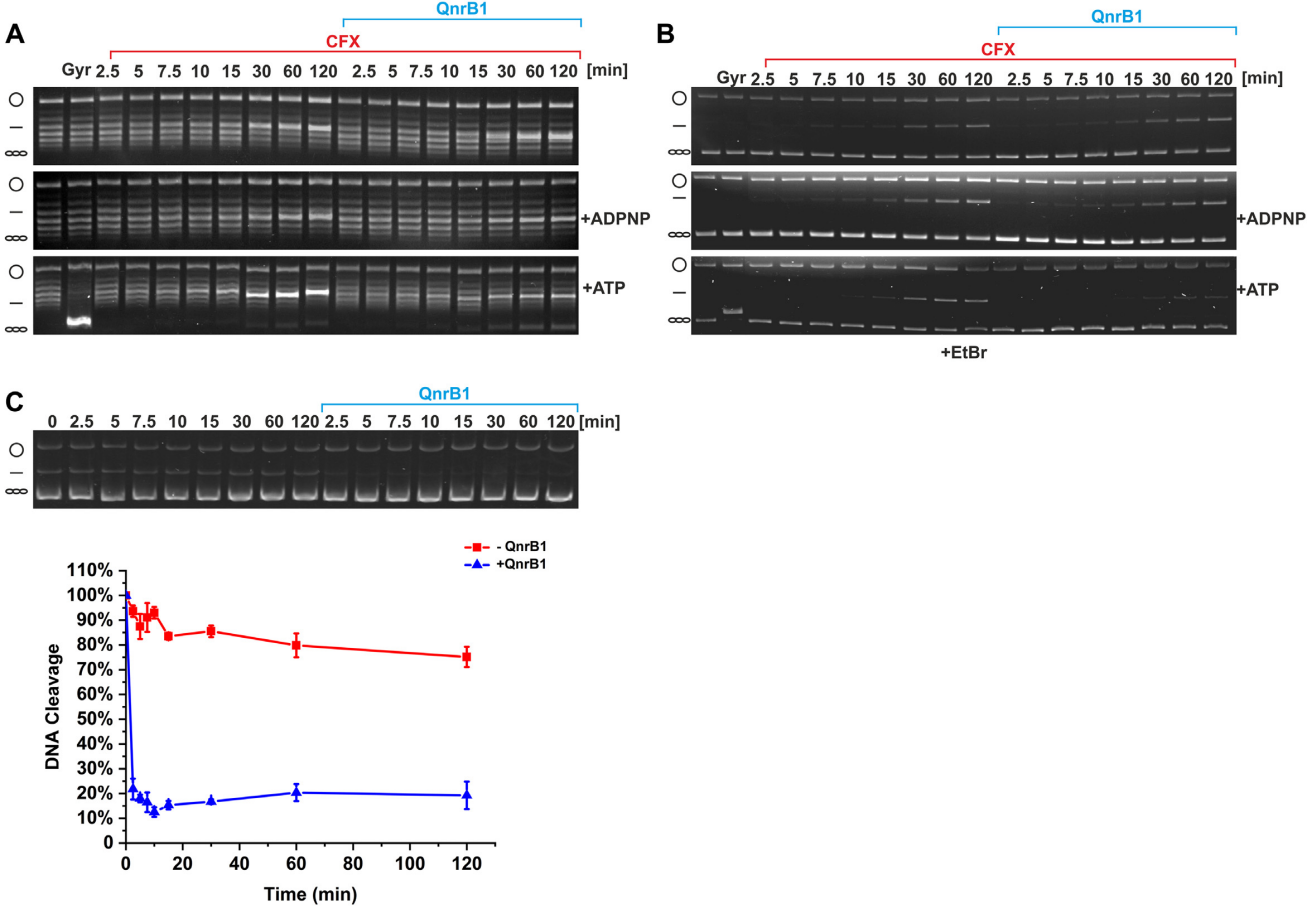
QnrB1 requires ATP hydrolysis to rescue DNA gyrase. (**A**) Time courses of DNA cleavage. The reactions contained 5 μM ciprofloxacin and 5 μM QnrB1 (as indicated) and were run without nucleotide, with ADPNP or ATP. After completion, the reactions were run on a gel without EtBr. (**B**) Same reactions run in the presence of EtBr. The amount of cleaved DNA is reduced when 5 μM QnrB1 is present and if ATP was added to the reaction. (**C**) Cleavage complex stability determined in a presence of 5 μM QnrB1. Initial DNA cleavage reactions were with 80 U of gyrase and 20 μM ciprofloxacin and were incubated for 10 min at 37°C to reach equilibrium and then diluted 20-fold with reaction buffer with or without 5 μM QnrB1. (*Top*) The samples were run on an EtBr gel. (*Bottom*) Linear DNA was quantified and plotted. Level of DNA cleavage at time 0 was set to 100%. Error bars represent the SD of at least three independent experiments.

G-segment binding and cleavage generally do not require ATP, and thus a hypothetical mechanism of protection, in which PRPs prevent drug binding or promote dissociation of bound drug from the complex, does not require the presence of the nucleotide. However, when we performed cleavage assays without a nucleotide, we found that the CFX-induced cleavage was not reversed by QnrB1 (Supplementary Figure S5A). Therefore, in order to act, QnrB1 requires either ATP binding, or ATP hydrolysis and subsequent ‘resetting’ (42) of the enzyme. To choose between these scenarios for QnrB1, we performed time-course experiments to closely monitor CFX-dependent cleavage complex formation in three different conditions: in the presence of ATP, its non-hydrolysable analogue ADPNP or in the absence of nucleotide. To give QnrB1 an opportunity to bind to gyrase before nucleotide-induced N-gate dimerization, nucleotides were added to the mixture last, following preincubation of gyrase and QnrB1. Addition of QnrB1 had no effect on cleavage in presence of ADPNP or when nucleotide was omitted; however, in the presence of ATP, a decrease in linear DNA was readily observed, allowing more complete supercoiling (Figure 3A, B).

If QnrB1 merely prevents cleavage complex formation (based on G-segment DNA mimicry or similar mechanism), it must interact with gyrase before the G-segment is cleaved and covalently bound to the enzyme. Conversely, if it promotes dissociation of or repositioning of the drug molecules to allow DNA re-ligation, it must be able to revert already existing cleavage complexes. We tested if QnrB1 can destabilize preformed cleavage complexes, consisting of gyrase, DNA and CFX. Figure 3C shows that in the presence of ATP and QnrB1, the cleaved DNA was quickly resealed whereas without QnrB1, the complex was stable for at least 2 h. No dissociation was observed without the nucleotide or with ADPNP (not shown). Thus, QnrB1 is able to interact with gyrase *after* cleavage complex formation to rescue the stalled enzyme and this process requires ATP hydrolysis.

### Protective effects of QnrB1 do not depend on strand passage

We showed that the inhibition of cleavage complex formation by QnrB1 depends on ATP hydrolysis by gyrase. We identified two possible mechanisms that can explain such behaviour: either PRP requires the energy of ATP hydrolysis and associated large-scale conformational changes to remove bound drug, or ATP-driven strand passage provides PRP with access to a temporarily exposed binding pocket within the enzyme. The strand passage requirement was suggested previously for MccB17 and toxin CcdB (38,43). In these two cases strand passage, occurring during ATP-independent relaxation of negatively supercoiled DNA, allowed for the binding of toxins.

We have investigated if QnrB1 can restore ATP-independent relaxation of negatively supercoiled DNA inhibited by CFX. No protection was observed (Supplementary Figure S5A) but instead the enzyme was additionally inhibited, suggesting that QnrB1 is still able to interact with gyrase. Surprisingly, QnrB1 on its own not only did not inhibit ATP-independent relaxation (Supplementary Figure S5BC) but slightly promoted it. To further test the requirement for strand passage, we addressed relaxation activities of A59_2_/B_2_ and A_2_/B47_2_ gyrase complexes mentioned above. In both cases rescue of ATP-dependent (A59_2_/B_2_) or ATP-independent (A_2_/B47_2_) relaxation from CFX was not observed and the enzyme appeared additionally inhibited by QnrB1 (Supplementary Figure S6AB). However, the effects of QnrB1 on its own on ATP-dependent and ATP-independent relaxation were different: while A59_2_/B_2_ relaxation was again clearly inhibited, relaxation by A_2_/B47_2_ was stimulated akin to the results with the full-length enzyme (Supplementary Figure S6CD).

In summary, these observations led us to consider that strand passage on its own is not important for QnrB1 activity. Therefore, we hypothesize that QnrB1 interacts with the ATPase domains of GyrB, which allosterically leads to the loss of the drug.

### QnrB1 stimulates gyrase ATPase activity

**Figure 4.**
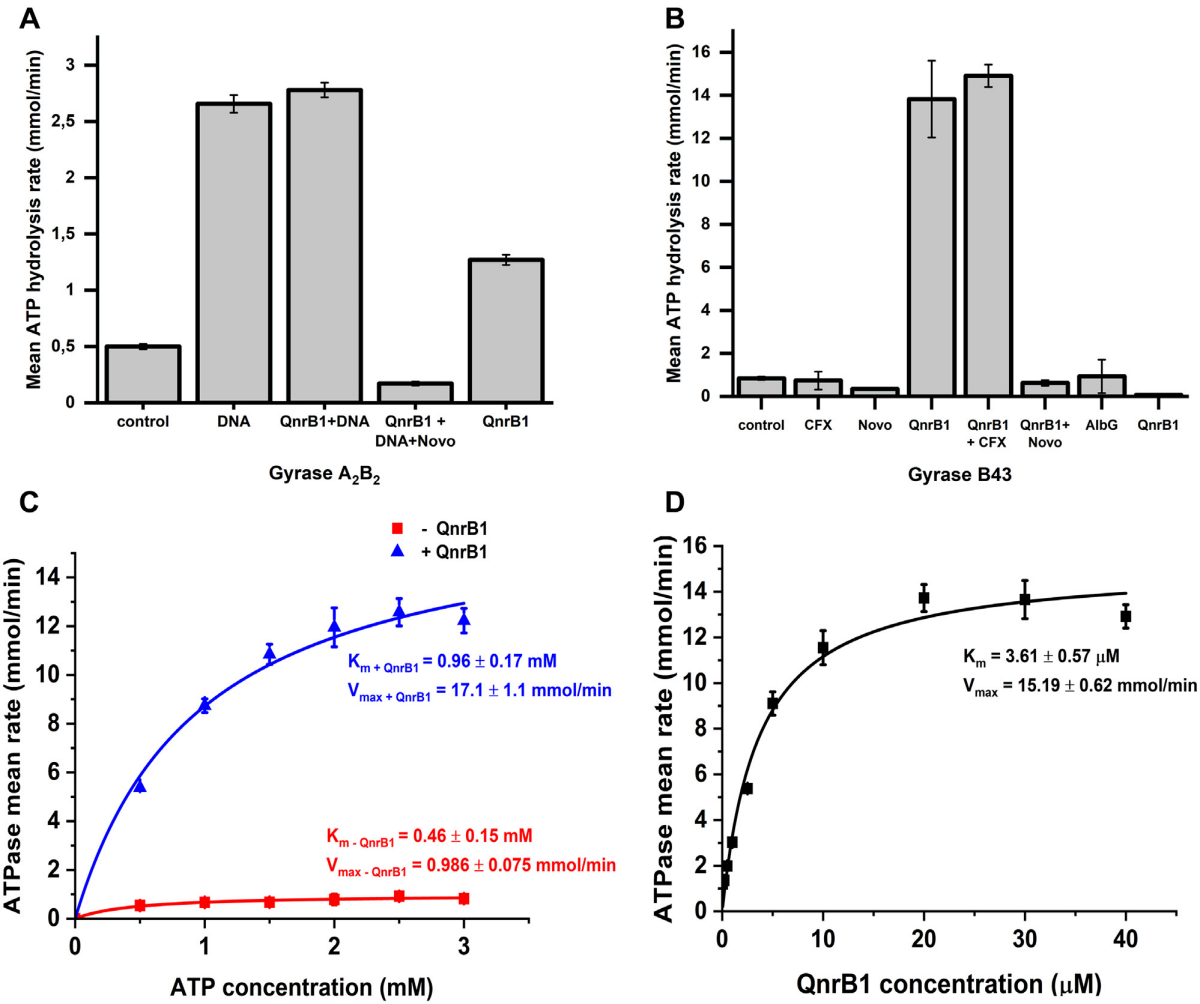
QnrB1 stimulates gyrase ATPase activity. (**A**) ATPase rate data for 50 nM gyrase (A_2_B_2_) complex mixed with: 10 nM DNA; 10 nM DNA and 5 μM QnrB1; 10 nM DNA, 5μM QnrB1 and 50 μM novobiocin; 5 μM QnrB1. (**B**) ATPase rate data for 4 μM GyrB43 mixed with QnrB1, drugs and DNA as indicated. DNA was used at 10 nM, novobiocin (Novo) at 50 μM, CFX at 5 μM and QnrB1 at 5 μM. (**C**) ATPase rate data and Michaelis-Menten fits for reactions conducted with different concentrations of ATP with (*blue*) or without (*red*) 5 μM QnrB1 for GyrB43. (**D**) ATPase assays data and Michaelis-Menten fit for reactions with 4 μM GyrB43 conducted with different concentrations of QnrB1 at constant concentration of ATP (1 mM). For all plots, error bars are expressed as the standard deviation of three independent experiments for the rate (mmol/min).

We proceeded to test if QnrB1 can stimulate DNA-independent and DNA-stimulated ATPase activity of gyrase. Figure 4A shows that QnrB1 indeed increased the ATP hydrolysis rate about 3-fold in the absence of DNA while the DNA-stimulated rate was not affected. Strikingly, when tested with isolated GyrB43 subunit, stimulation became much stronger (Figure 4BCD). The ATPase reaction rate of GyrB43 with 5 μM QnrB1 was ∼17 times higher than the rate with no QnrB1 present (*V*_max (– QnrB1)_ = 0.986 ± 0.075 mmol/min vs *V*_max (+ QnrB1)_ = 17.1 ± 1.1 mmol/min). Loop deletion mutant QnrB1 ΔTTR stimulated ATPase activity equally to the WT variant (Supplementary Figure S7).

Obtained *K*_m_ values for ATP in QnrB1-stimulated GyrB43 reactions lie in the same order of magnitude as values for non-stimulated reactions (*K*_m – QnrB1_ = 0.46 ± 0.15 mM, *K*_m + QnrB1_ = 0.96 ± 0.17 mM), suggesting that the addition of QnrB1 at this concentration does not affect ATP binding (Figure 4C). QnrB1-dependent activation of GyrB43 ATPase activity seemed to follow Michaelis–Menten kinetics with *K*_m_ = 3.61 ± 0.57 μM and *V*_max_ = 15.19 ± 0.62 mmol/min. (Figure 4D). Experiments with full-length GyrB subunit showed very weak (<2-fold) but reproducible activation (not shown).

### QnrB1 binds to the GyrB subunit *in vitro*

**Figure 5.**
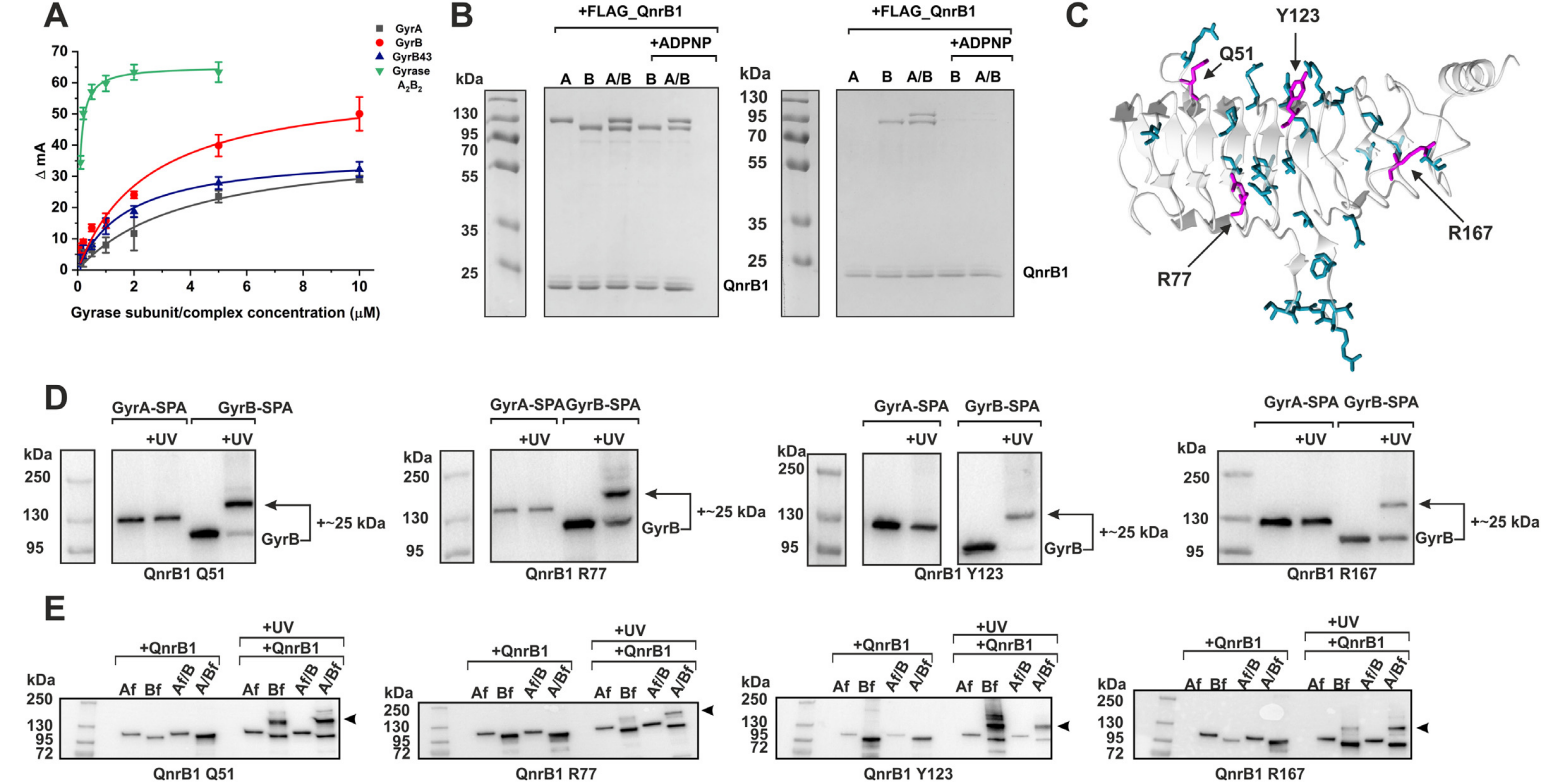
Interactions between GyrB and QnrB1. (**A**) Fluorescence anisotropy assay showing binding of individual gyrase subunits and A_2_B_2_ complex to QnrB1. Gray square – GyrA; red circle – GyrB; blue triangle – GyrB43; green triangle – gyrase A_2_B_2_ complex. ΔmA indicates the change in anisotropy (in milliunits). Error bars represent the SD of three independent experiments. (**B**) *In vitro* pull-down assay with N-terminally FLAG-tagged QnrB1 and purified GyrA, GyrB and A_2_B_2_ complex. *Left* – input, *right* – pull-down (eluates from M2 (α-FLAG) agarose). A Coomassie stained SDS-PAGE gel is shown. (**C**) A cartoon representation of QnrB1 monomer (grey, PDB: 2XTW) is shown with residues chosen for crosslinking experiments displayed as dark cyan cylinders. Residues that produce crosslinks when replaced with *p*Bpa are marked with arrows and displayed as magenta cylinders. (**D**) *In vivo* crosslinking anti-FLAG western blots of QnrB1 Q51, R77, Y123 and R167 *p*Bpa variants to chromosomally encoded GyrA-SPA and GyrB-SPA in *E. coli*. Visible band-shifts correspond to an increase in molecular weight of ∼25 kDa, roughly equivalent to QnrB1. (**E**) *In vitro* crosslinking anti-FLAG western blots of QnrB1 Q51, R77, Y123 and R167 *p*Bpa variants with different FLAG-tagged gyrase subunits and mixtures of both tagged and untagged subunits (GyrA/3xFLAG-GyrB or GyrA-FLAG/GyrB) (Af - C-terminally FLAG GyrA; Bf - N-terminally 3xFLAG GyrB). Crosslinks are indicated by an arrow.

We sought to determine whether GyrB43 ATPase domain is indeed involved in QnrB1 binding. We measured interactions between gyrase and QnrB1 using a fluorescence anisotropy-based assay, similarly to the work done previously for gyrase regulator YacG (32). All gyrase subunits show some binding to QnrB1. As can be seen from Figure 5A, N-terminally labeled [Alexa488]-QnrB1 binds to both GyrB43 and full-length GyrB with estimated *K*_d,GyrB43_ of 1.77 ± 0.22 μM and *K*_GyrB_ 2.53 ± 0.74 μM respectively. However, the binding for GyrB did not reach full saturation even at >10 μM [GyrB] (higher concentrations could not be tested due to the increasing viscosity). Gyrase A_2_B_2_ complex bound QnrB1 with *K*_d_ of 0.08 ± 0.01 μM. Finally, the highest *K*_d_ of 3.9 ± 1.2 μM was obtained for the isolated GyrA subunit. Preincubation of subunits with ADPNP decreased the binding: QnrB1 bound to GyrB with *K*_d_ of 3.8 ± 1.2 μM and to GyrB43 with *K*_d_ = 12.2 ± 2.7 μM. The most significant drop in affinity was observed for A_2_B_2_ complex where *K*_d_ increased >20-fold to 1.78 ± 0.31 μM (Supplementary Figure S8), suggesting that QnrB1 cannot interact with the ‘restrained’ GyrB conformation that is stabilized by the nucleotide analog (44).

We used an additional technique (pull-down) to confirm these findings. We found that N-terminally FLAG-tagged QnrB1 was able to bind purified GyrB, both independently and in the context of the GyrA_2_B_2_ complex (Figure 5B). GyrA on its own did not interact with FLAG-QnrB1, but it was retained on the resin in the presence of GyrB, suggesting that gyrase complex is not disrupted by QnrB1 binding. When reactions were pre-incubated with ADPNP, no binding was observed. In the reverse pull-down experiment (Supplementary Figure S9), QnrB1 was retained in the presence of 3xFLAG-GyrB, 3xFLAG-GyrB/GyrA complex or GyrB/GyrA-FLAG complex. Once again, preincubation with ADPNP completely abolished binding. We have tried to detect interactions of QnrB1 with smaller parts of GyrB (i.e. ATPase-transducer GyrB43, ATPase GyrB24 and TOPRIM GyrB47) but only full-length GyrB could pull-down QnrB1 (Supplementary Figure S10).

In summary, FA and pull-down experiments show that QnrB1 can interact with both GyrA and GyrB subunits but the interaction with GyrB is much stronger.

### QnrB1 crosslinks to GyrB *in vivo* and *in vitro*

We next used an orthogonal photo-crosslinkable amino acid benzoyl-phenylalanine (*p*Bpa) to investigate the QnrB1-gyrase interactions in intact *E. coli* cells (34,45). To pinpoint which residues of QnrB1 are likely to be involved, we selected several residues using the published crystal structure (PDB:2XTW). The ConSurf web server (46) was used to aid picking potential residues. Non-conserved residues were not taken into consideration as there is low possibility of their importance in the interaction. Highly conserved residues with an obvious structural role and buried residues were also excluded. By application of this approach, 29 surface-exposed QnrB1 residues were selected and replaced with the *p*Bpa (Supplementary Table S1 and Figure 5C). *E. coli* DY330 derivatives, in which chromosomal GyrB or GyrA genes are fused with SPA purification tags (GyrA-SPA and GyrB-SPA) (27,47) were used as hosts for expression of QnrB1 *p*Bpa variants. Four residues (Q51, R77, Y123, R167) produced crosslinks, all of them with GyrB (Figure 5D, Supplementary Table S1 and Supplementary Figures S11-13). All crosslinked residues were found on one face of QnrB1 (Face 2), suggesting a defined interaction interface (Figure 5C).

In order to confirm these results, crosslinking experiments were repeated *in vitro* with purified Q51*p*Bpa, R77*p*Bpa, Y123*p*Bpa and R167*p*Bpa QnrB1 variants and FLAG-tagged *E. coli* gyrase subunits described above. Q51*p*Bpa and Y123*p*Bpa were found to crosslink strongly to GyrB while R77*p*Bpa and R167*p*Bpa gave weaker crosslinks also to GyrB (Figure 5E). The molecular weight of the main crosslinked band corresponded to the attachment of a single QnrB1 molecule to GyrB. Additional shifted species were observed as weaker bands, corresponding to the attachment of two QnrB1 molecules. Interestingly, while the double crosslink band was very strong for GyrB-Y123*p*Bpa, it completely disappeared in case of the GyrA/B complex, suggesting that only the single crosslink band is biologically relevant.

Additional experiments with Q51*p*Bpa and Y123*p*Bpa QnrB1 variants and purified GyrB subdomains showed that QnrB1 was approximately stoichiometrically crosslinked to GyrB and to the GyrB43 domain (Supplementary Figure S14) but did not interact with a shorter GyrB24 domain or TOPRIM (GyrB47) (though a very weak band was detected for TOPRIM-Y123*p*Bpa). To further prove the specificity of observed crosslinks, we carried out competition experiments, where increasing amounts of WT QnrB1 were added to the crosslinking reactions with GyrB and QnrB1 Y123*p*Bpa or GyrB43 and QnrB1 Y123*p*Bpa (Supplementary Figure S15). In both cases, WT QnrB1 easily replaced QnrB1 Y123*p*Bpa, with 10x excess of unlabeled protein almost completely preventing crosslinking.

Following the results of FA and pull-down experiments where ADPNP was found to block the GyrB-QnrB1 interaction, we tested the effects of ADPNP on the observed crosslinks. GyrB43 and full-length GyrB were pre-incubated with ADPNP to induce subunit dimerization and crosslinked to QnrB1 Y123*p*Bpa. ADPNP largely prevented crosslinking of full-length GyrB subunit with QnrB1 (Supplementary Figure S16A) though it did not influence the crosslink efficiency of GyrB43 (Supplementary Figure S16B). Finally, the QnrB1 ΔTTR mutant (Y123pBpa Δ_107-109_) was equally well crosslinked to GyrB (Supplementary Figure S16C).

## DISCUSSION

### Inhibitory effects of QnrB1 on supercoiling and relaxation reactions of DNA gyrase

Topoisomerase-interacting PRPs have been proposed to be gyrase regulators with different activities. MfpA, Qnr and EfsQnr were all reported to both protect *E. coli* gyrase from quinolone inhibition and at the same time inhibit gyrase supercoiling activity. In this work, we show that QnrB1 requires a concentration >10 000-fold higher than the enzyme for efficient inhibition of supercoiling, whilst 100 times less QnrB1 is required to effectively relieve FQ inhibition.

Apart from supercoiling, DNA gyrase is capable of ATP-independent relaxation of negatively supercoiled DNA, where strand passage is believed to proceed in the reverse direction (bottom to top). Strikingly, this reaction was not inhibited by QnrB1 at any concentration (in fact, we consistently observed slight stimulation of relaxation activity at the highest concentrations of QnrB1 - see Supplementary Figure S5). Similarly, ATP-independent relaxation of negatively supercoiled DNA by the A_2_/B47_2_ complex was not inhibited but rather stimulated by high doses of QnrB1 (Supplementary Figure S6). An A59_2_/B_2_ complex with truncated C-terminal domains can carry out ATP-dependent relaxation when the strand passage is thought to occur top to bottom from N-gate to the C-gate (40). This reaction was strongly inhibited by QnrB1. Taken together, these results suggest that QnrB1 is only able to inhibit gyrase reactions which require normal top to bottom strand passage, coupled with ATP hydrolysis. This inhibition might result from the decreased DNA binding by the enzyme in presence of PRP, and indeed the main assumption of the ‘G-segment DNA mimicry’ hypothesis is that PRPs act as competitive DNA binders leading to inhibition of the enzyme. QnrB1 indeed decreased DNA binding in fluorescence anisotropy (FA) and gel retardation (EMSA) experiments, but the effect was divorced from the protective effect, being visible only at concentrations of QnrB1 ∼10-fold higher (IC_50_ = ∼11 μM). Other protein inhibitors, directly occluding the DNA binding site on gyrase, such as YacG are reported to have apparent *K*_i_ as low as 35 nM (32). Moreover, in the presence of CFX which stabilizes DNA binding, QnrB1 was unable to outcompete DNA.

Overall, we found that QnrB1 does not inhibit the normal function of gyrase unless at very high concentrations, does not inhibit the ATP-independent process of DNA relaxation and does not act as a good G-segment DNA mimic.

### Interaction of QnrB1 with GyrB and GyrB43

Both QnrB1 and MfpA were reported to bind *E. coli* gyrase in native gel retardation (24) and surface plasmon resonance (19) experiments, respectively. Models were proposed in which PRPs bind to the positively charged GyrA dimer (19,22). In more recent pull-down experiment (48), GST-tagged QnrB1 protein was shown to bind *E. coli* GyrB stronger than GyrA. Likewise, in two-hybrid system experiments the QnrB1 interaction signal for GyrB was 7- to 11-fold higher than the signal for GyrA (49).

Our study presents overwhelming evidence that the main binding partner of QnrB1 is GyrB and that GyrB43 forms at least a part of the binding interface. Association with GyrB is not only supported by FA, pull-down and crosslinking experiments but also by the observed stimulation of ATP hydrolysis, which is particularly strong in the case of GyrB43 (17-fold). The difference in the magnitude of the QnrB1 effect (only modest 3-fold stimulation was observed for the full-length gyrase) might be attributed to the much higher baseline ATPase activity, exhibited by the full-length GyrB subunit, compared to GyrB43 (we used 4 μM of GyrB43 versus 50 nM of full-length subunits to achieve comparable reaction rates). QnrB1 cannot stimulate the ATPase activity of full-length gyrase to the same extent as DNA and therefore is unlikely to bind to the GyrB in exactly the same way. One possibility is that QnrB1 stabilizes the GyrB43 dimer and thus promotes ATP hydrolysis.

In all three types of binding experiments, we performed (FA, pull-downs, crosslinking) pre-incubation of GyrB, GyrB43 or A_2_B_2_ complex with ADPNP prevented or reduced QnrB1 binding. Based on the results of cross-linking experiments, we propose that the QnrB1 dimer makes extensive contacts along a significant portion of GyrB, interacting with both GyrB43 and TOPRIM in a way that at least a part of QnrB1 enters the cavity formed by TOPRIM domains. GyrB dimerization (as promoted by ADPNP) leads to steric clashes and precludes binding. However, crosslinking of QnrB1 to GyrB43 was not affected by the presence of nucleotide analogue, meaning that at least a part of the interacting surface is still available in the GyrB43 dimer. We propose that the ‘open’ conformation (50,51) of gyrase, stabilized by FQs, likely allows for QnrB1 binding, yet QnrB1 cannot access its binding site when the N-gate is ‘locked’ by ADPNP. We also cannot exclude that GyrB adopts a new conformation, not seen previously in crystal structures, in order to bind QnrB1.

Despite we believe GyrB constitutes the main binding interface, we should point out that weaker (*K*_d_ = 3.9 μM) binding was also observed for GyrA, the finding which corresponds well to the two-hybrid system data (49). We will try to reconcile these observations below.

### Loops are essential for protection, but not for binding

A 12-amino acid loop, protruding from the rod-like QnrB1 scaffold, was shown to be the main determinant of protection against FQs (20,23), with a deletion of three amino acids (107–109, TTR) completely abolishing protection (23). A similar structural element has been found in AlbG (12). In this study we for the first time analyzed the role of the AlbG loop and found that similarly to QnrB1, its deletion abolished protection against albicidin *in vivo*. However, when we characterized properties of QnrB1 ΔTTR was found that it bound to GyrB equally strongly as WT protein in pull-down and crosslinking experiments and activated ATPase activity to the same extent. QnrB1 ΔTTR also inhibited gyrase supercoiling activity to the same extent as WT protein and decreased DNA binding to the gyrase. Interestingly, in reported two-hybrid system experiments, loop deletions did not perturb the interaction with GyrB (49). The same work has shown that GyrA interaction depends on the presence of the QnrB1 loops and is affected by sublethal doses of FQs. We propose that the loop might interact with GyrA and is important for the precise positioning of the PRP required to perturb bound drug to allow religation of DNA, but is not by itself the main driver of binding.

### Models for QnrB1 protection mechanism

The suggestion that PRPs are DNA mimics is attractive, but the original ‘G-segment’ DNA mimicry model is not supported by our and others’ data (20). Such a model predicts a reduction in DNA binding by gyrase that would reduce susceptibility to all agents that stabilize cleavage complexes involving the G-segment-gyrase interface. However, Qnr does not protect against the proteinaceous gyrase poison CcdB (52) nor against the natural product simocyclinone D8, which binds to the GyrA subunit in the ‘saddle’ region and prevents G-segment DNA binding. Moreover, QnrB1 was found to act synergistically with simocyclinone (53). In our work, we show that the activity of three different PRPs, QnrB1, AlbG and McbG show a high level of specificity toward three different gyrase poisons. Further, the cleavage inhibition activity of the PRP strictly required ATP hydrolysis by gyrase, ruling out any models where PRPs are expected to simply bind to the enzyme and block the gyrase poison binding site.

Given the significant structural similarities between PRPs, it is reasonable to expect that the overall mechanism of topoisomerase protection should be universal but can also account for the observed toxin-specificity. Moreover, it should account for the two clearly distinct modalities of QnrB1 action (general interaction with the enzyme via GyrB subunit and drug-specific protection, likely mediated by loop elements and potentially involving GyrA).

We have previously hypothesized (26) that topoisomerase targeting PRPs function via a T-segment mimicry mechanism, in which they are captured and translocated through the enzyme in a manner analogous to the T-segment. In such a model PRP would presumably enter the inner cavity delineated by the GyrB43 subunits, in a manner analogous to the short DNA duplex found in a crystal structure of *S. pneumoniae* Topo IV (PDB:5J5Q). In this position PRP would not be expected to make any contacts with the TOPRIM domain, and its binding should not be affected by the GyrB dimerization. The results of FA, pull-down and crosslinking experiments performed in this work, contradict this notion: while ADPNP ‘locking’ does not completely prevent GyrB43–QnrB1 interaction (as the crosslink is still observed), the K_d_ is markedly increased and all interactions with full-length GyrB are completely abolished. Therefore, for this model to remain valid, QnrB1 (and by extension other PRPs), while mimicking a T-segment in terms of capture and, potentially passage through the enzyme, would have to differ from a T-segment in the details of their interactions with GyrB, namely to bind in a way that allows them to interact with both GyrB43 and TOPRIM domains of GyrB. Interactions with TOPRIM are supported by the results of A_2_/B47_2_ relaxation assays, where the effects of QnrB1 presence can be consistently seen both with and without FQs.

**Figure 6.**
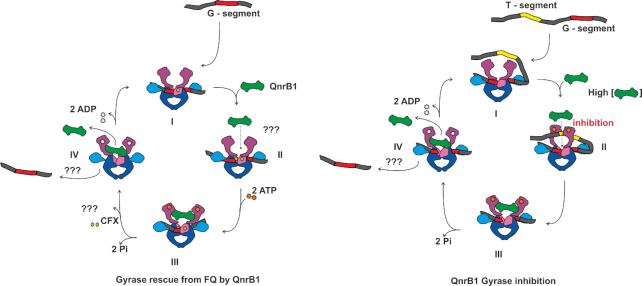
Potential mechanism of action of QnrB1. *Gyrase rescue from FQ by QnrB1:***I**. DNA gyrase cleavage complex formation. **II**. QnrB1 initial binding to the ATP-operated clamp (ATPase and transducer domains) in GyrB. **III, IV**. After ATP binding and hydrolysis, specific (loop-mediated) QnrB1 interaction results in fluoroquinolone removal and subsequent release of the PRP. This might be accompanied by the DNA release. *QnrB1 gyrase inhibition*: **I**. DNA gyrase with bound G segment. **II**. At high concentrations, QnrB1 competes with the T-segment and binds to the ATP-operated clamp, preventing T-segment binding. **III, IV**. After ATP hydrolysis, QnrB1 is released, which might be accompanied by the release of DNA.

Taking our results together and considering previous models, we are able to propose an alternative poisoned complex recognition model for QnrB1 action which incorporates some elements of the T-segment DNA mimicry (Figure 6) and suggest that the same scheme applies to all topoisomerase-interacting PRPs. In this model, to bind to gyrase in physiological conditions (i.e. *without* FQ present) QnrB1 must engage with GyrB and outcompete T-segments, which requires a very high excess of QnrB1 over gyrase and is unlikely to happen. However, at high concentrations QnrB1 may interfere with normal top to bottom strand passage and inhibits supercoiling and ATP-dependent relaxation (Figure 6, ‘Gyrase inhibition’). The same interference helps to promote ATP-independent relaxation, which is thought to occur in the reverse direction, i.e. DNA entering from the C-gate. Inhibition of top to bottom strand passage thus helps to shift the thermodynamic relaxation equilibrium toward more relaxed DNA species. Binding of QnrB1 to GyrB also reduces DNA binding observed in EMSAs and FA assays.

In the model, FQ-mediated cleavage complex stabilisation allows QnrB1 to readily interact with the enzyme by removing competition with the T-segment. The protective activity of PRPs strictly requires ATP hydrolysis by gyrase, moreover, QnrB1 binding actively promotes ATP hydrolysis. We can consider a protective mechanism involving formation of a QnrB1-stabilized conformation for which the gyrase poison has low affinity. To be effective, such a conformation must have lower energy than the toxin-stabilized one that it replaces. Energy input is then required for subsequent QnrB1 release, otherwise the enzyme would be constantly inhibited by the QnrB1 itself. Therefore, the requirement for the energy input in the form of ATP is logical. We suggest that QnrB1 act as ‘spoke in the wheel’ which, upon ATP hydrolysis can be ‘pushed’ through the enzyme to physically dislodge bound poison or causes allosteric changes leading to the same effect (Figure 6, ‘Gyrase rescue from FQ’). This dislodgement is poison-specific and requires specific amino acids in loops of QnrB1. Analogous mechanisms are observed for ribosome protective factors such as TetO and TetM which hydrolyze GTP in order to release themselves from the ribosome (54).

Further clarification of this model requires knowledge of fine molecular details of interactions between PRPs, including QnrB1, and their targets. The recent development of a cryo-EM platform for structural studies of the *E. coli* gyrase complex (55) has the potential to address such challenges. New structural information may allow to design gyrase inhibitors based on PRP peptides, novel antibacterials which avoids PRP-driven resistance or small molecules which will block the PRP-gyrase interactions, to keep the potency of existing drugs such as FQs.

## DATA AVAILABILITY

The data that support the findings of this study are presented in the main text and in the online Supplementary Data. Additional data can be made available from authors upon reasonable request.

## Supplementary Material

gkaa1266_Supplemental_File

## Notes

Present address: Mikhail Metelev, Department of Cell and Molecular Biology, Uppsala University, Uppsala, Sweden.
