# Supplementary material for: Pentapeptide repeat protein QnrB1 requires ATP hydrolysis to rejuvenate poisoned gyrase complexes
Source: Nucleic Acids Res. 2021 Jan 12;49(3):1581–96. doi: 10.1093/nar/gkaa1266 (PMC7897471; doi:10.1093/nar/gkaa1266)
Supplement: gkaa1266_Supplemental_File [file gkaa1266_supplemental_file.pdf]

Supplementary Materials

Includes Supplementary Figures S1-S17 and Supplementary Tables S1-S3.

Łukasz Mazurek[1,†], Dmitry Ghilarov[1,†,], Elizabeth Michalczyk[1], Zuzanna Pakosz[1,2], Mikhail Metelev[3,&], Wojciech Czyszczoń[1], Karolina Wawro*[1], *Iraj Behroz[4], Svetlana Dubiley[3], Roderich D. Süssmuth[4] and Jonathan G. Heddle[1,*]*

[1]Malopolska Centre of Biotechnology, Jagiellonian University, Krakow, Poland

[2]Postgraduate School of Molecular Medicine, Warsaw, Poland

[3]Institute of Gene Biology, Moscow, Russia

[4]Institute of Biological Chemistry, Technische Universität Berlin, Berlin, Germany

**These authors contributed equally*

[&]*Present address:* Department of Cell and Molecular Biology, Uppsala University, Uppsala, Sweden

[5]*Correspondence needs to be addressed to:*

dmitry.gilyarov@uj.edu.pl

jonathan.heddle@uj.edu.pl

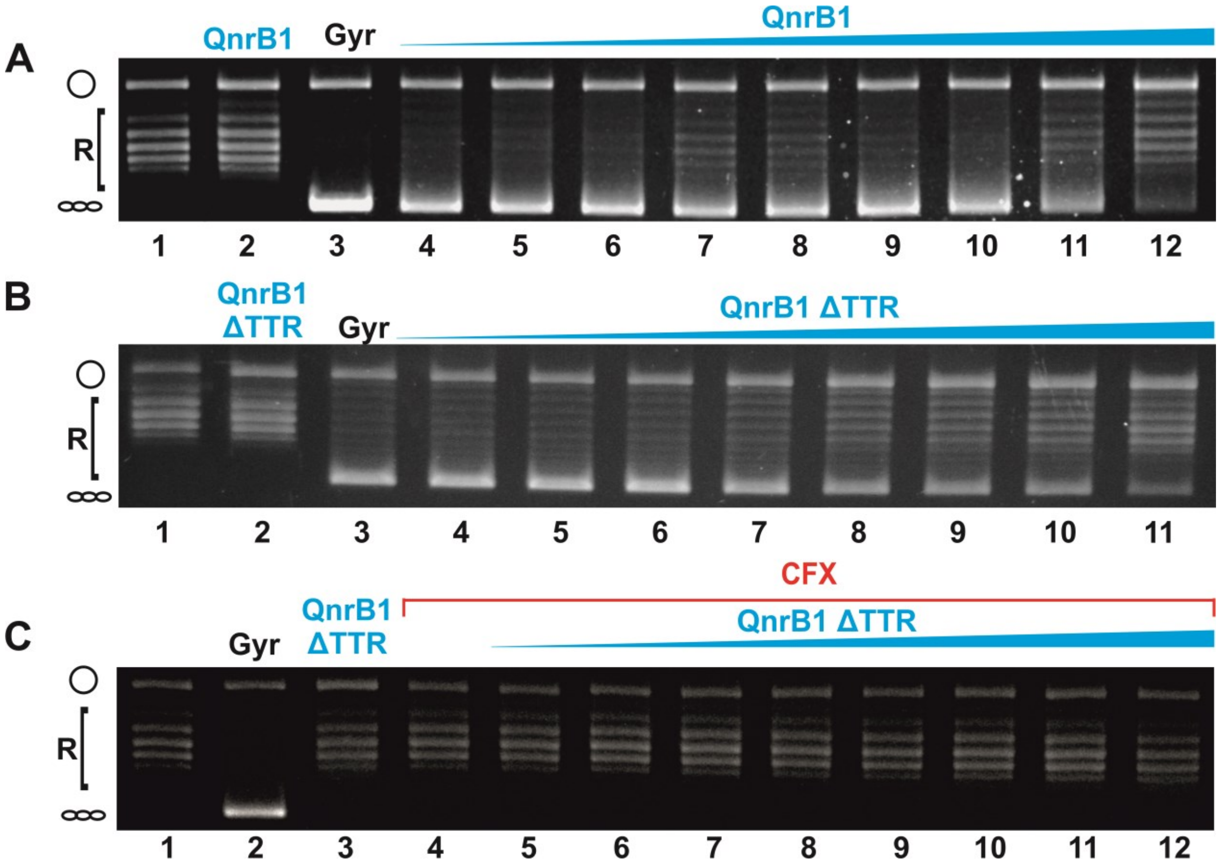

Supplementary Figure S1. Inhibition of gyrase supercoiling reaction by the increasing amounts of QnrB1. (**A**) Plasmid supercoiling assay showing the inhibitory effect of high concentrations of QnrB1. Lane 1: relaxed pBR322, lane 2: no nuclease activity was observed upon addition of 50 µM QnrB1, Lane 3: gyrase and relaxed pBR322 lanes 4-12: effect of increasing concentration of QnrB1 on gyrase supercoiling activity (0.0016; 0.008; 0.04; 0.2; 1; 5; 10; 20; 40 µM). (**B**) Plasmid supercoiling assay showing inhibitory effect of high concentrations of QnrB1 ΔTTR. Lane 1: relaxed pBR322, lane 2: nuclease control (pBR322+50 µM QnrB1 ΔTTR); lane 3: gyrase and relaxed pBR322; lanes 4-11: effect of increasing concentration of QnrB1 on gyrase supercoiling activity (0.0016; 0.008; 0.04; 0.2; 1; 5; 10; 25; 50 µM). (**C**) Plasmid supercoiling assay showing lack of protection by QnrB1 ΔTTR. Lane 1: relaxed pBR322; lane 2: nuclease control (pBR322+50 µM QnrB1 ΔTTR); lane 3: gyrase, 1 U; lane 4: gyrase and 5 µM CFX; lanes 5-12: gyrase and 5 µM CFX and increasing concentration of QnrB1 ΔTTR (0.0016; 0.008; 0.04; 0.2; 1; 5; 10; 25; 50 µM).

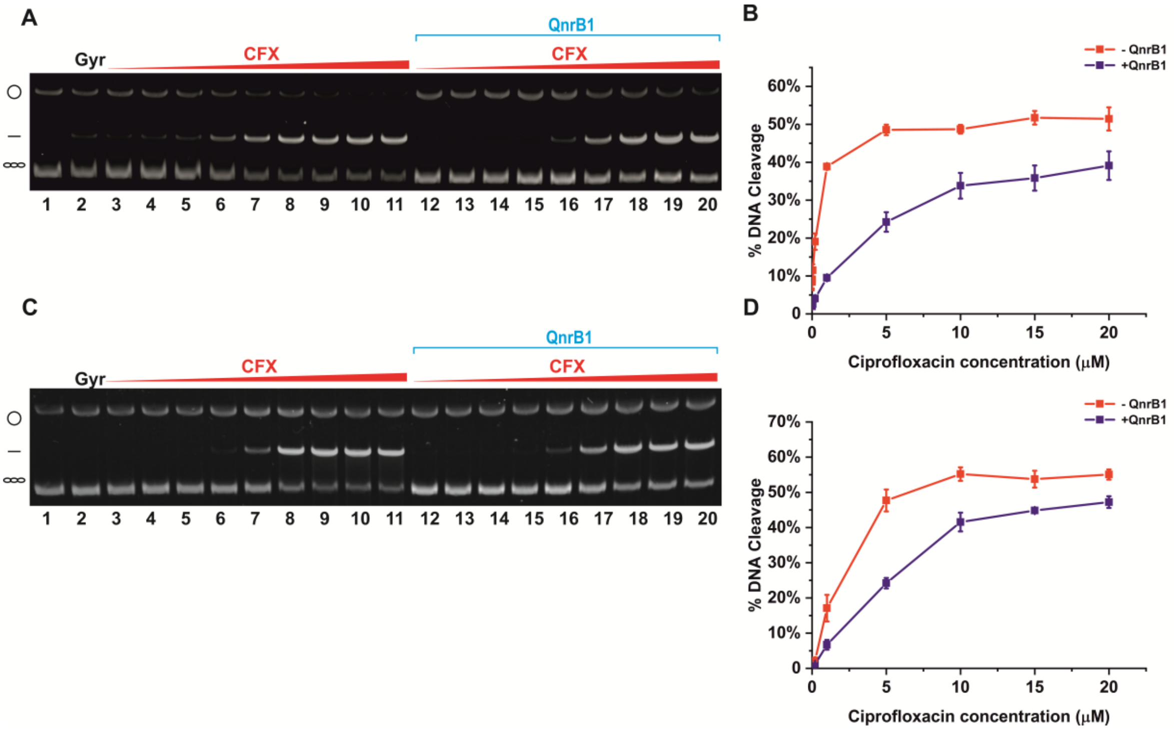

Supplementary Figure S2. Inhibition of ciprofloxacin-induced gyrase cleavage activity by QnrB1. (**A**) DNA cleavage assay with gyrase A_2B_2 complex. Lane 1: negatively supercoiled pBR322; lane 2: negatively supercoiled pBR322 and 5 U (20 nM) A_2B_2; lanes 3 – 11: effect of increasing concentration of ciprofloxacin on DNA cleavage (0.0016; 0.008; 0.04; 0.2; 1; 5; 10; 15; 20 μM). Lanes 12 – 20: effect of increasing concentration of ciprofloxacin on DNA cleavage (same concentrations) in the presence of 5 μM QnrB1. (**B**) Graph showing dependence of DNA cleavage (percent) from ciprofloxacin concentration for A_2B_2 with (purple squares) and without (red squares) QnrB1. Error bars represent standard deviation (SD) of 3 independent experiments. (**C**) DNA cleavage assay with gyrase $A59_2B_2$ complex. Lane 1: negatively supercoiled pBR322; lane 2: negatively supercoiled pBR322 and 20 nM gyrase $A59_2B_2$ complex; lanes 3 – 11: effect of increasing concentration of ciprofloxacin on DNA cleavage (0.0016; 0.008; 0.04; 0.2; 1; 5; 10; 15; 20 μM). Lanes 12 – 20: effect of increasing concentration of ciprofloxacin on DNA cleavage (0.0016; 0.008; 0.04; 0.2; 1; 5; 10; 15; 20 μM) in presence of 5 μM QnrB1. (**D**) Graph showing dependence of DNA cleavage (percent) from ciprofloxacin concentration for $A59_2B_2$ with (purple squares) and without (red squares) QnrB1. Error bars represent standard deviation (SD) of 3 independent experiments.

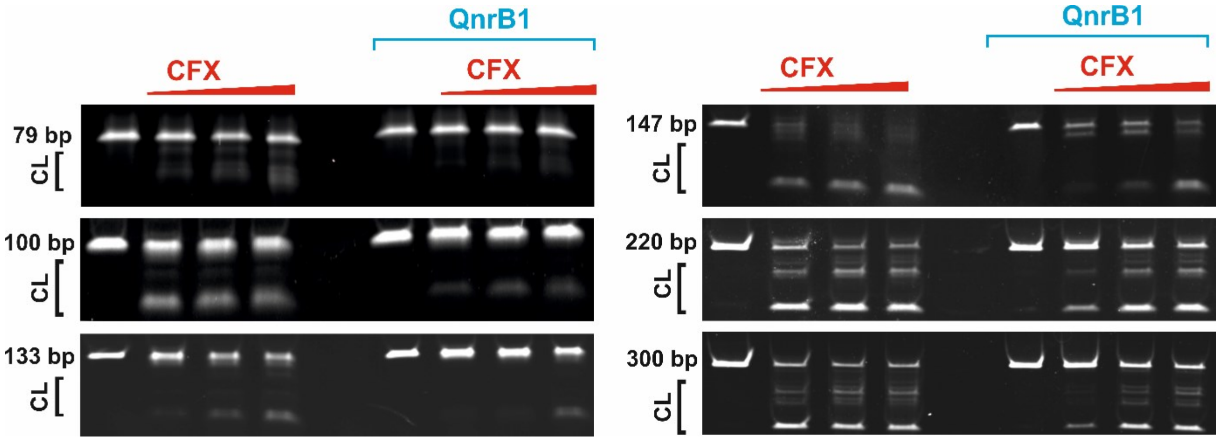

Supplementary Figure S3. **Cleavage of short DNA fragments by DNA gyrase and protection by QnrB1**. Length of fragments (in base pairs) is indicated at the left. CL – cleaved DNA. 50 nM gyrase complex is present in each reaction. QnrB1 (5 µM) and CFX (0.1, 1, 10 µM) were added as indicated.

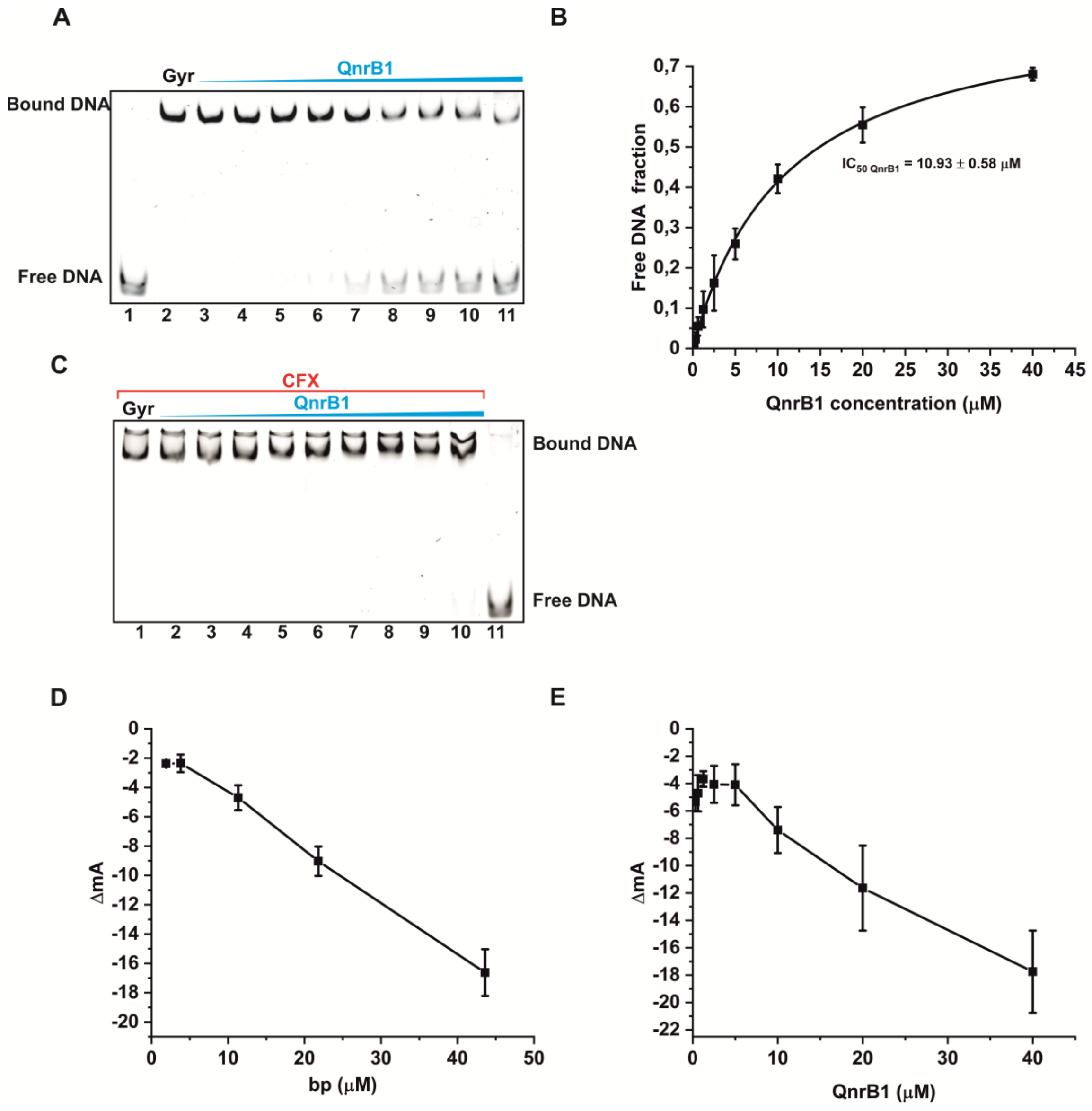

Supplementary Figure S4. Effects of QnrB1 on DNA binding by gyrase. (**A**) EMSA showing the effect of increasing concentration of QnrB1 on DNA binding. Shown is SYBR Gold-stained 6% TBM gel. Lane 1: free 147 bp DNA; lane 2: gyrase (200 nM) added to DNA; lanes 3-11: effect of increasing concentrations (0.2; 0.3; 0.6; 1.3; 2.5; 5; 10; 20; 40 μM) of QnrB1 on DNA binding by gyrase. (**B**) Graph showing dependence of free DNA fraction on concentration of QnrB1. IC_{50} value for QnrB1 is calculated from the fitted curve. Error bars are expressed as the standard deviation of three independent experiments. (**C**) Effect of increasing concentration of QnrB1 on DNA binding in a presence of 5 μM CFX. Lane 1: gyrase, DNA and 5 μM CFX; lane 2-10: effect of increasing concentration (0.2; 0.3; 0.6; 1.3; 2.5; 5; 10; 20; 40 μM) of QnrB1 on DNA binding by DNA gyrase; lane 11: free DNA. (**D**) Fluorescence anisotropy measurement of QnrB1 displacement by increasing concentration of linear DNA (linear pBR322). 50 nM Alexa-488-QnrB1 and 1 μM DNA gyrase complex was used in

reaction. Concentrations of linear DNA calculated as concentration of base pairs (bp) were as follows: (2; 4; 11; 22; 44 μM). (**E**) Fluorescence anisotropy measurement of DNA displacement by increasing concentration of QnrB1. 20 nM 90 bp DNA-Cy5 and 1 μM DNA gyrase complex was used in reaction. Concentrations of QnrB1 were as follows: (0.3125; 0.625; 1.25; 2.5; 5; 10; 20; 40 μM).

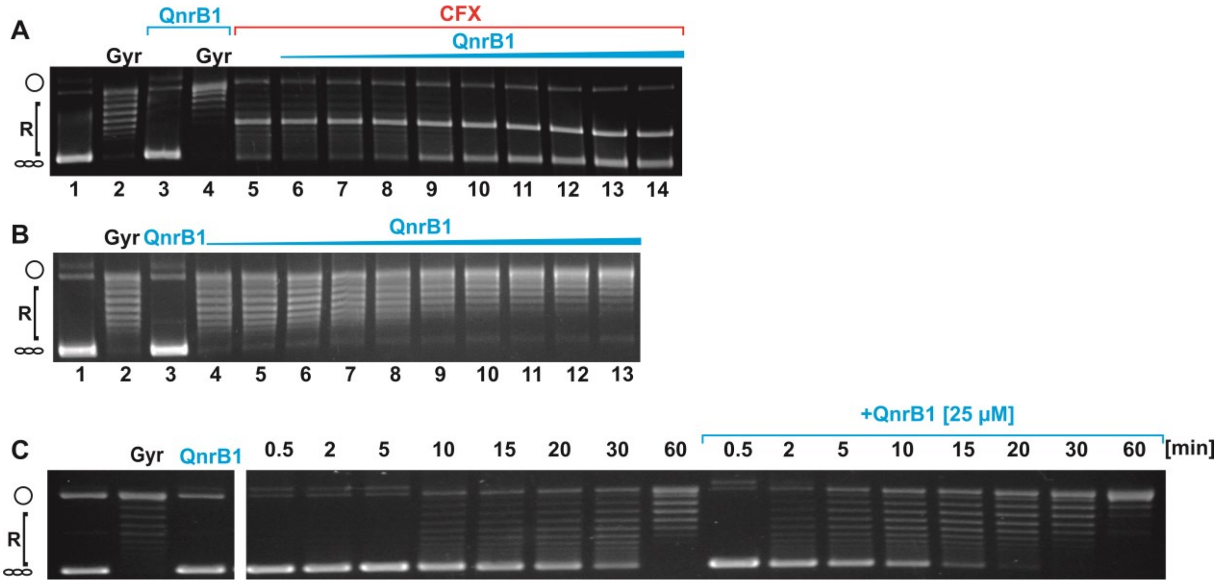

Supplementary Figure S5. **Effect of QnrB1 on ATP-independent relaxation and cleavage of negatively supercoiled DNA. (A)** Relaxation of negatively supercoiled DNA in a presence of ciprofloxacin and increasing QnrB1 concentrations. Samples were treated with SDS and proteinase K to release cleaved DNA. Lane 1: negatively supercoiled pBR322; lane 2: relaxation by 20 nM gyrase; lane 3: lack of detectable nuclease activity in the purified QnrB1 (50 µM QnrB1); lane 4: relaxation in the presence of 50 µM QnrB1; lanes 5-14: DNA relaxation in the presence of CFX (5 µM) and (6-14) the effect of increasing concentrations of QnrB1 (0.008; 0.04; 0.2; 1; 5; 10; 20; 25; 50 µM). (**B**) Relaxation of negatively supercoiled DNA in presence of increasing QnrB1 concentrations. Lane 1: negatively supercoiled pBR322; lane 2: relaxation by gyrase; lane 3: lack of detectable nuclease activity in the purified QnrB1 (50 µM QnrB1); lanes 4-13: relaxation in presence of increasing concentrations of QnrB1 (0.0016, 0.008; 0.04; 0.2; 1; 5; 10; 20; 25; 50 µM). **(C)** Time course of DNA relaxation in presence of QnrB1. Gyr: DNA and gyrase after 60 minutes. QnrB1: nuclease control (50 µM QnrB1+DNA). Time points are indicated above the lanes.

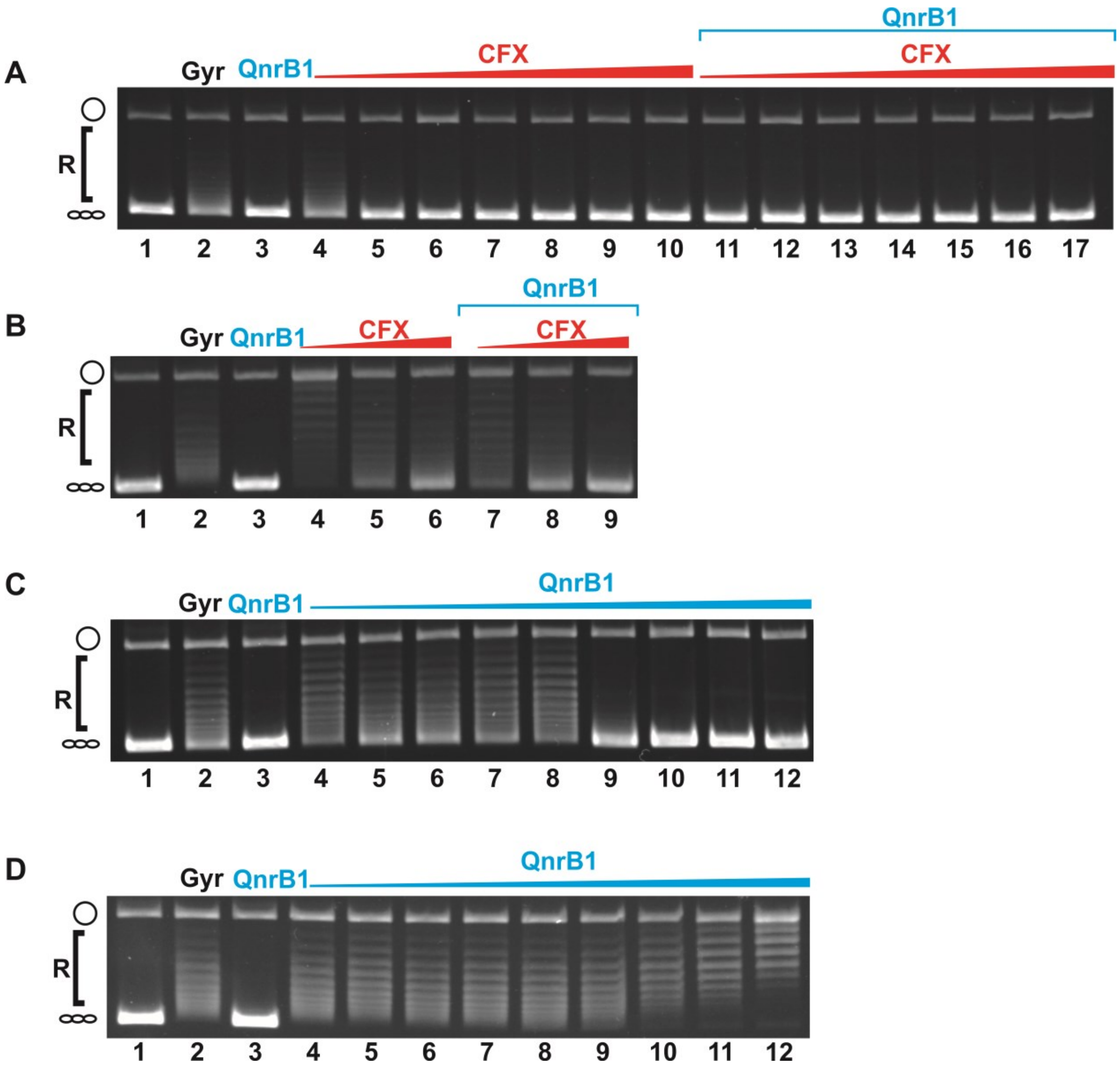

Supplementary Figure S6. Effects of QnrB1 on relaxation by truncated gyrase enzymes: ATP-dependent (by $A59_2/B_2$) and ATP-independent (by $A_2/B47_2$). (**A**) $A59_2/B_2$ ATP-dependent relaxation assay in presence of increasing concentration of ciprofloxacin and 5 µM QnrB1. Lane 1: negatively supercoiled pBR322; lane 2: relaxation by $A59_2/B_2$; lane 3: relaxation in the presence of 50 µM QnrB1; lanes 4-10: DNA relaxation in the presence of increasing concentrations of CFX (0.2; 1; 5; 10; 20; 25; 50 µM); lanes 11-17: DNA relaxation in the presence of increasing concentrations of CFX (as before) and 5 µM QnrB1. (**B**) $A_2/B47_2$ ATP-independent relaxation assay in the presence of increasing concentrations of ciprofloxacin and 5 µM QnrB1. Lane 1: negatively supercoiled pBR32; lane 2: relaxation by $A_2/B47_2$; lane 3: relaxation in the presence of 50 µM QnrB1; lanes 4-6: DNA relaxation in the presence of increasing concentrations of CFX (20; 35; 50 µM) ; lanes 7-9: DNA relaxation in the presence of increasing concentrations of CFX (as before) and 5 µM QnrB1. (**C**) $A59_2/B_2$ ATP-dependent

relaxation assay in the presence of increasing concentrations of QnrB1. Lane 1: negatively supercoiled pBR322; lane 2: relaxation by $A59_2/B_2$; lane 3: relaxation in the presence of 40 μM QnrB1; lanes 4-12: DNA relaxation in the presence of increasing concentrations of QnrB1 (0.0016; 0.008; 0.04; 0.2; 1; 5; 10; 20; 40 μM). (**D**) $A_2/B47_2$ ATP-independent relaxation assay in the presence of increasing concentrations of QnrB1. Lane 1: negatively supercoiled pBR322; lane 2: relaxation by $A_2/B47_2$; lane 3: relaxation in the presence of 40 μM QnrB1; lanes 4-12: DNA relaxation in the presence of increasing concentrations of QnrB1 (0.0016; 0.008; 0.04; 0.2; 1; 5; 10; 20; 40 μM)

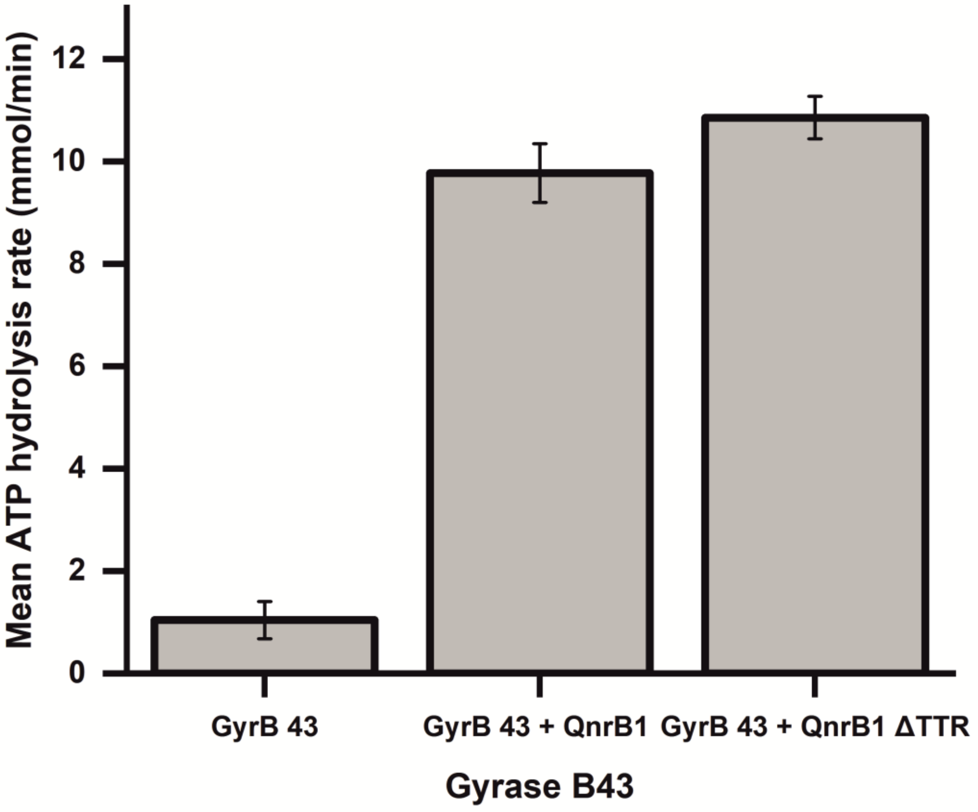

Supplementary Figure S7. ATPase rate data for QnrB1 ΔTTR. ATPase rate data for 4μM GyrB43 mixed with 5 μM QnrB1 and 5 μM QnrB1 ΔTTR. Error bars are expressed as the standard deviation of three independent experiments.

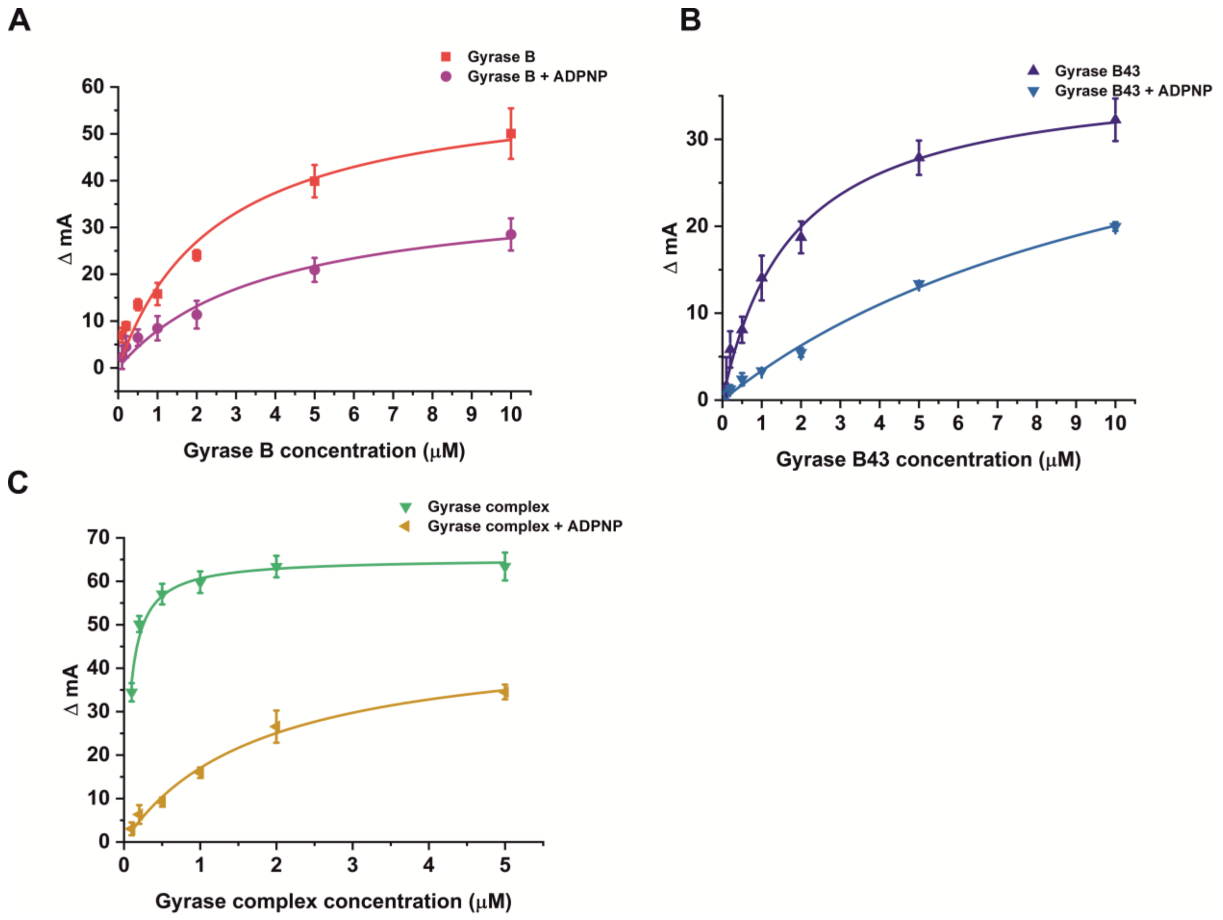

Supplementary Figure S8. Interaction of gyrase subunits with QnrB1 measured by fluorescence anisotropy. 50 nM Alexa-488-labelled QnrB1 was used in all experiments. (**A**) GyrB, $K_d = 2.53 \pm 0.74$ μM; $K_{d\,(+ADPNP)} = 3.8 \pm 1.2$ μM; (**B**) GyrB43, $K_d = 1.77 \pm 0.22$ μM, $K_{d\,(+ADPNP)}$ was 12.2 ± 2.7 μM; **(C)** Gyrase A_2B_2 complex, $K_d = 0.08 \pm 0.01$ μM, $K_{d\,(+ADPNP)} = 1.78 \pm 0.31$ μM. ΔmA indicates the change in anisotropy (in milliunits). Error bars are expressed as the standard deviation of three independent experiments.

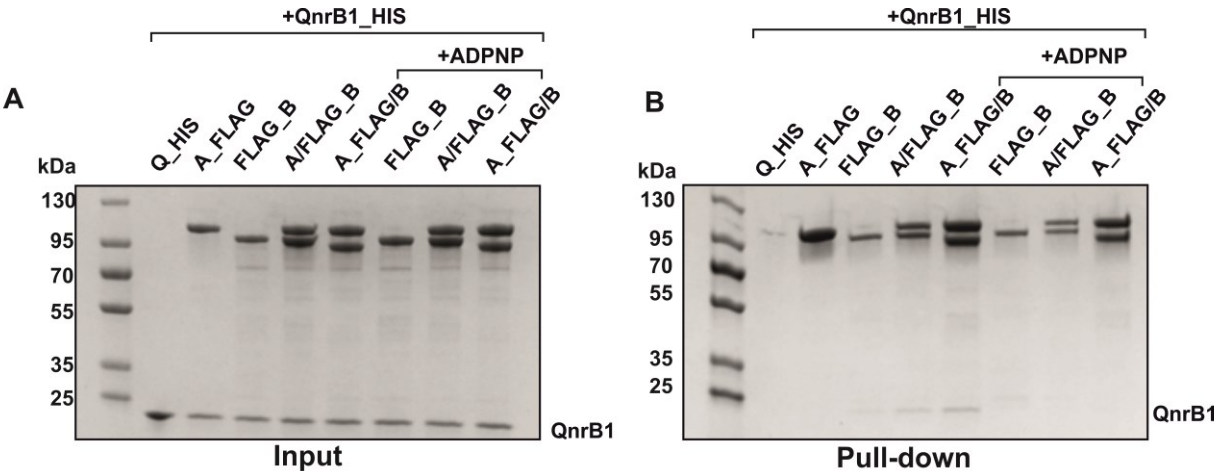

Supplementary Figure S9. SDS-PAGE gels showing reverse pull-down of QnrB1 and different gyrase subunits. 5 µM QnrB1 and 0.65 µM of gyrase subunit/complex were used in each reaction. Samples were pre-incubated with 1 mM ADPNP where indicated.

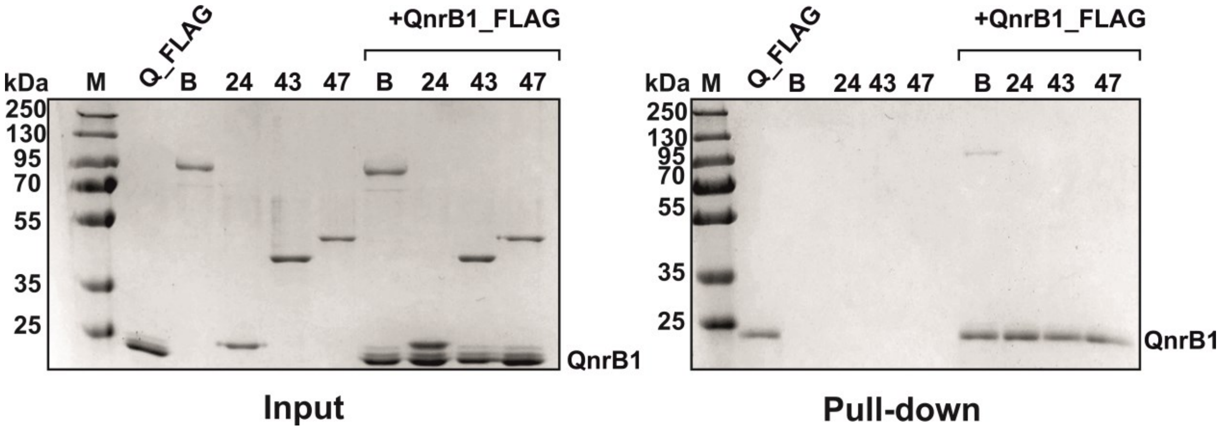

Supplementary Figure S10. SDS-PAGE gel showing pull-down with FLAG_QnrB1 and GyrB sub-domains. 5 µM QnrB1 and 0.65 µM of indicated gyrase subdomains were used in each reaction.

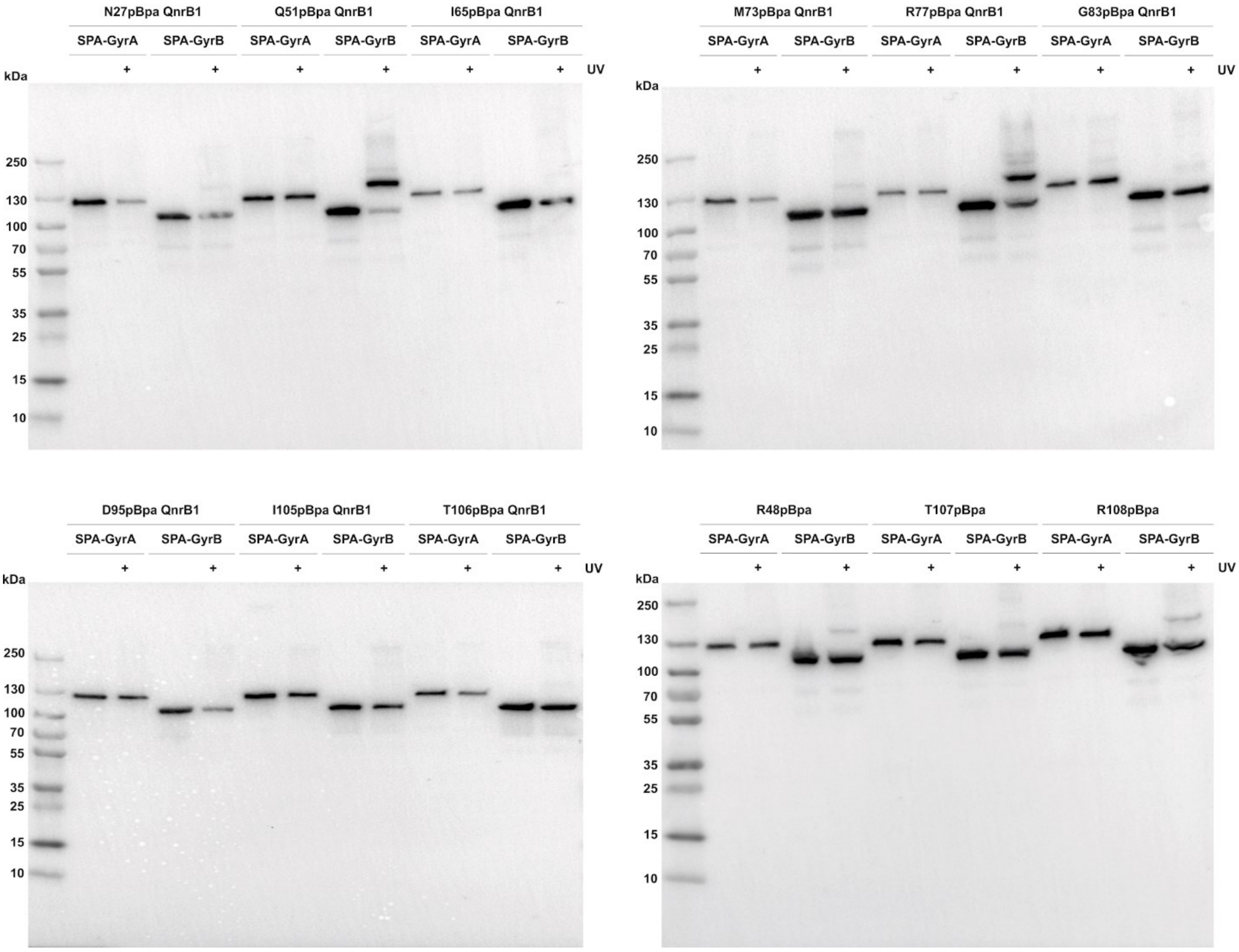

Supplementary Figure S11. Uncropped Western blots for *in vivo* crosslinking of QnrB1 mutants to GyrA-SPA and GyrB-SPA. Residues N27, Q51, I65, M73, R77, G83, D95, I105, T106, R48, T107, R108 are substituted to pBpa. Lanes with UV-treated cells are indicated by (+).

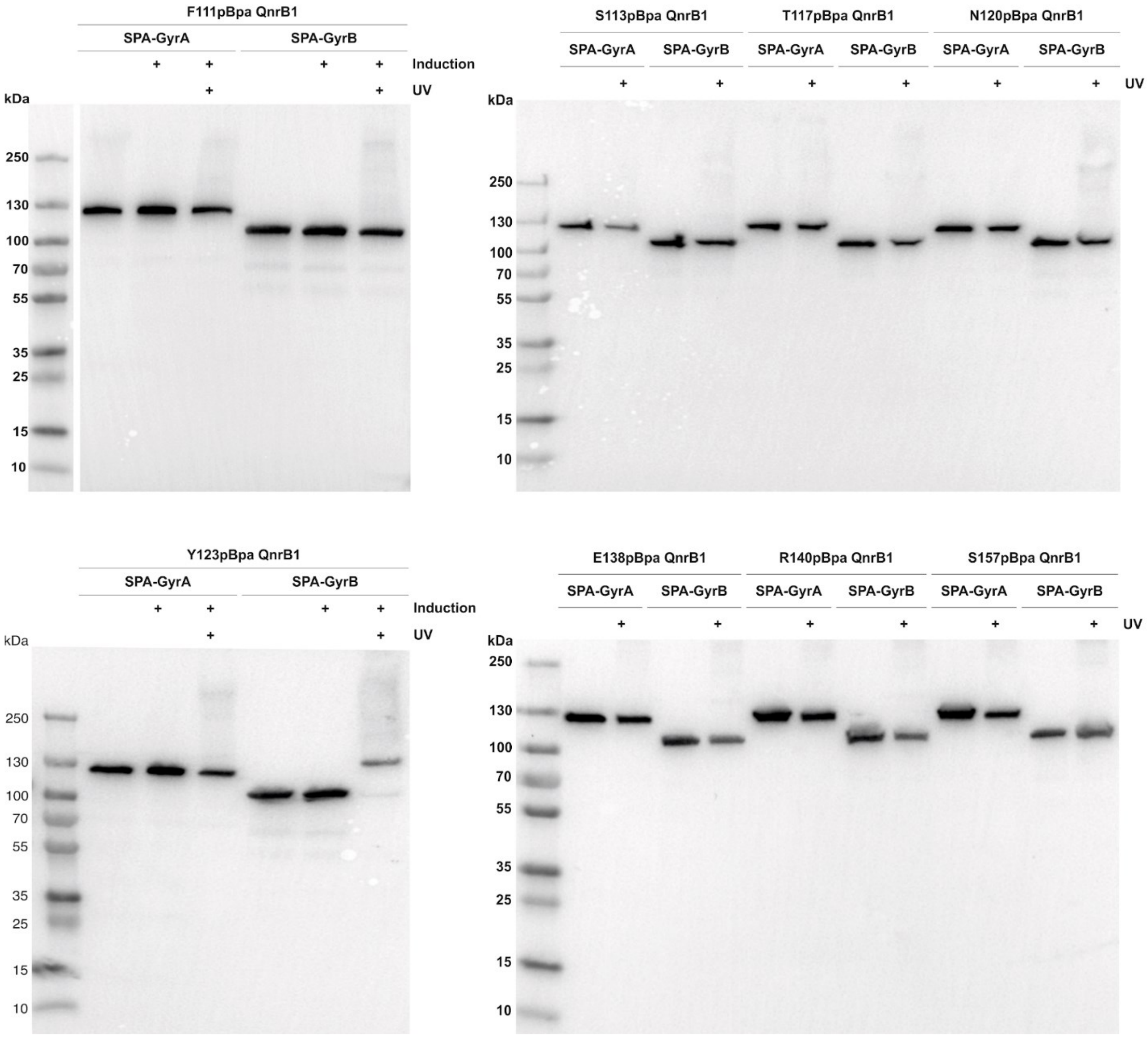

Supplementary Figure S12. Uncropped Western blots for *in vivo* crosslinking of QnrB1 mutants to GyrA-SPA and GyrB-SPA. Residues F111, S113, T117, N120, Y123, E138, R140, S157 are substituted to pBpa. Lanes with UV-treated cells are indicated by (+).

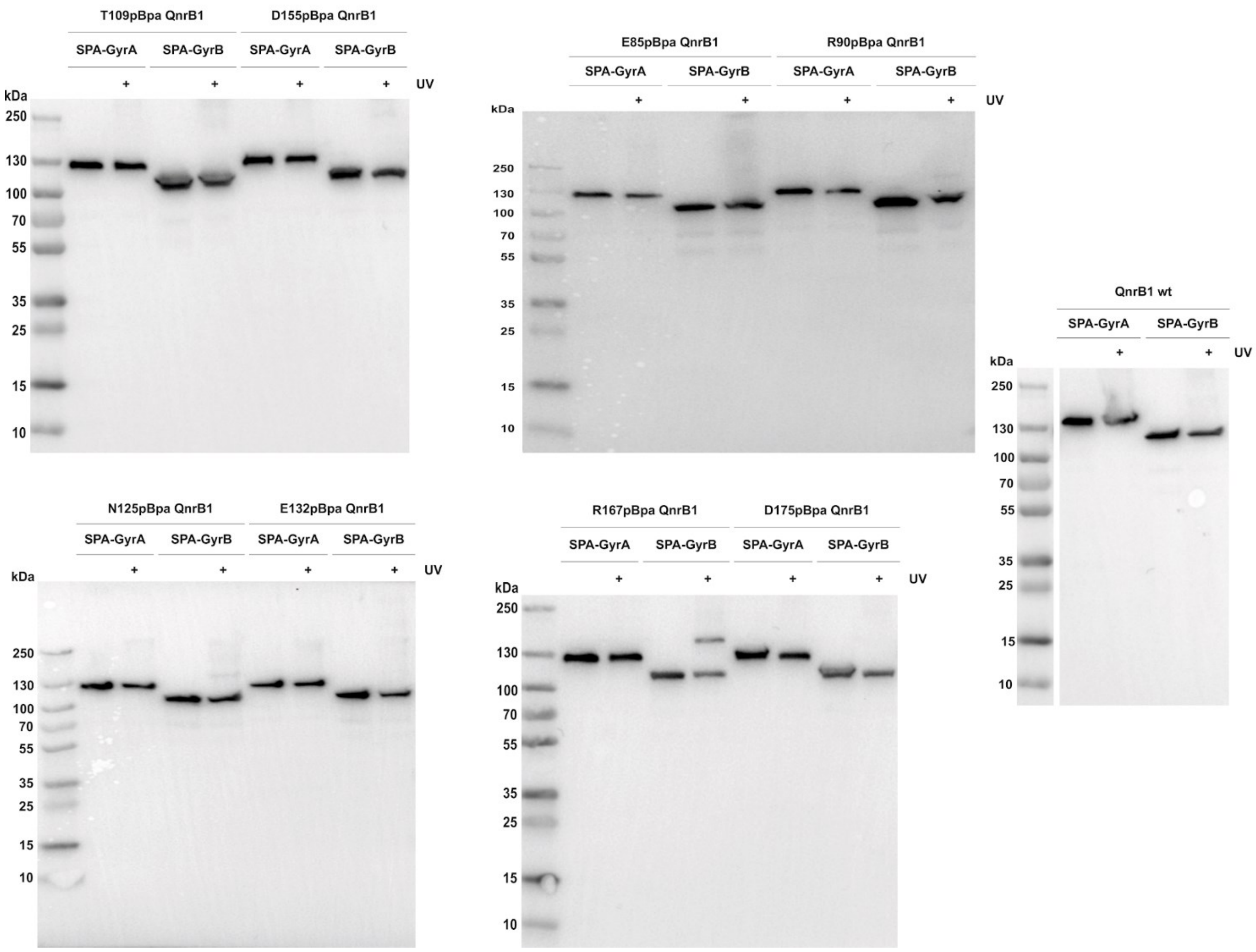

Supplementary Figure S13. Uncropped Western blots for in vivo crosslinking of QnrB1 mutants to GyrA-SPA and GyrB-SPA. Residues T109, D155, E85, R90, N125, E132, R167, D175 substituted to pBpa. Lanes with UV-treated cells are indicated by (+). Also shown is a negative control crosslinking with WT (unlabelled) QnrB1.

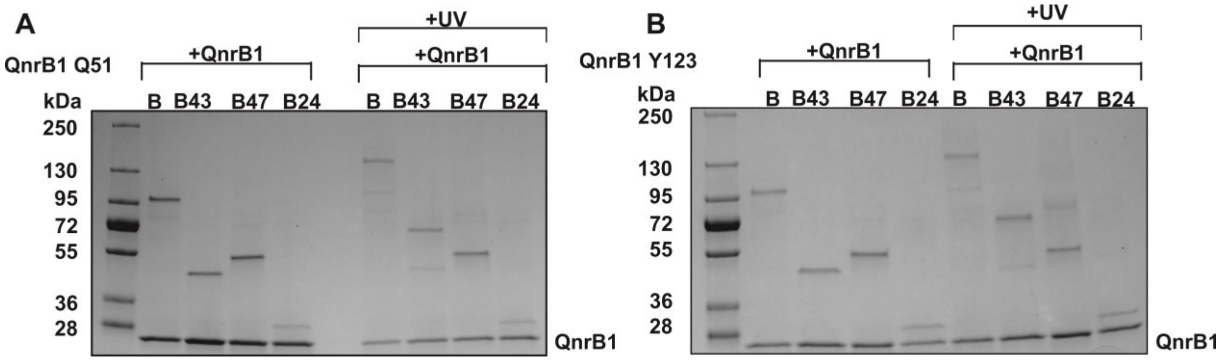

Supplementary Figure S14. SDS-PAGE gels showing UV – induced crosslinking of QnrB1 Q51 *p*Bpa and QnrB1 Y123 *p*Bpa with different gyrase subdomains. 5 µM of QnrB1 and 0.4 µM of indicated gyrase subunit was used in the reaction. (**A**) crosslink with QnrB1 Q51 *p*Bpa; (**B**) crosslink with QnrB1 Y123 *p*Bpa.

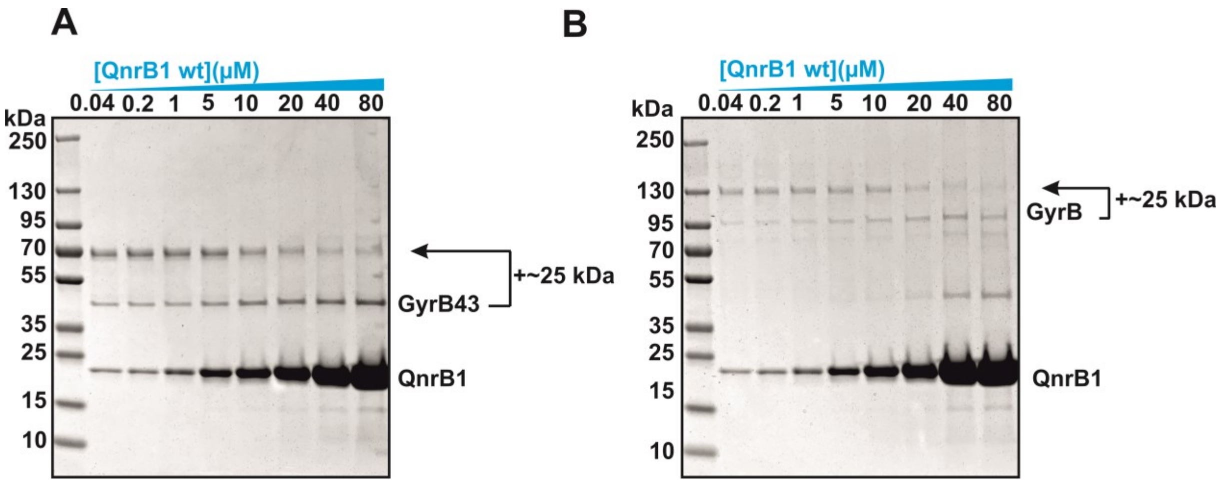

Supplementary Figure S15. Competition crosslinking experiment with QnrB1 wt and QnrB1 Y123*p*Bpa. (**A**) GyrB43 subunit. Each reaction consisted of 0.4 µM GyrB43, 5 µM QnrB1 Y123*p*Bpa and increasing concentration of WT unlabelled QnrB1 **(B)** GyrB subunit. Each reaction consisted of 0.4 µM GyrB, 5 µM QnrB1 Y123*p*Bpa and increasing concentrations of WT unlabelled QnrB1. Band-shifts corresponding to QnrB1 crosslinking are indicated. Coomassie-stained SDS-PAGE gels are shown.

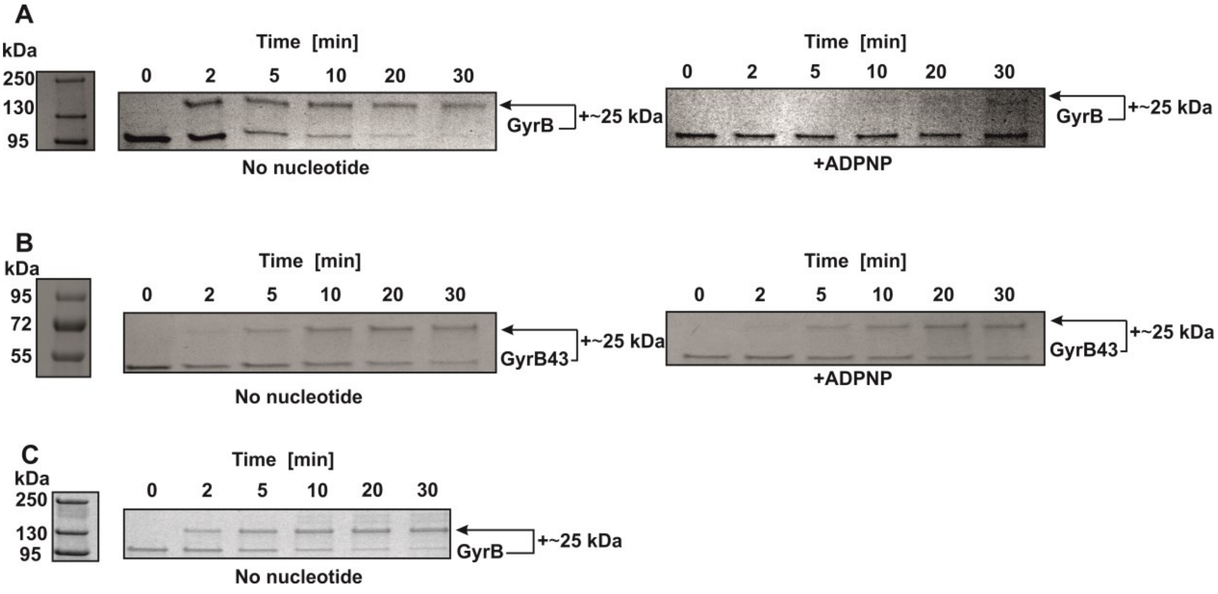

Supplementary Figure S16. Time course of *in vitro* crosslinking of GyrB and GyrB43 to QnrB1Y123pBpa. (**A**) Crosslink to gyrase B subunit with (*right*) and without (*left*) ADPNP. (**B**) Crosslink to gyrase B43 with (*right*) and without (*left*) ADPNP. (**C**) Crosslinking of QnrB1 Y123*p*Bpa ΔTTR mutant. Coomassie-stained SDS-PAGE gels are shown. Band-shifts corresponding to QnrB1 crosslinking are indicated.

Supplementary Table S1. List of QnrB1 residues replaced with *p*Bpa; **bold** are residues, which produced UV crosslinks

**QnrB1 position**	**PRP position** (1)
N27	i^{+2}
R48	loop A
**Q51**	loop A
I65	i^{-1}
M73	i^{+2}
**R77**	i^{+1}
G83	i^{+2}
E85	i^{-1}
R90	i^{-1}
D95	i^{-1}
I105	loop B
T106	loop B
T107	loop B
R108	loop B
T109	loop B
F111	loop B
S113	loop B
T117	i^{+1}
N120	i^{-1}
**Y123**	i^{+1}
N125	i^{-1}
E132	i^{+1}
E138	i^{+2}
R140	i^{-1}
D155	i^{-1}
S157	i^{+1}
**R167**	i^{+1}
D175	i^{-1}
R187	i^{+1}

Table S2. List of plasmids used in the study.

Name	Backbone	Source	Purpose
pBAD-*mcbG*	pBAD/His B	this work	Expression of untagged McbG protein
pBAD-*albG*	pBAD/His B	this work	Expression of untagged AlbG protein
pBAD-*albG* Δ91–97	pBAD/His B	this work	Expression of untagged AlbG loop deletion mutant
pBAD-*qnrb1*	pBAD/His B	this work	Expression of untagged QnrB1 protein
pET28- *mcbG*	pET-28a(+)	this work	Expression of His-tagged McbG protein
pET28-*albG*	pET-28a(+)	Gift of Dr. Mikhail Metelev (Uppsala University)	Purification of 6xHis-tagged AlbG protein
pET28- *qnrb1*	pET-28a(+)	Gift of Dr. Mikhail Metelev (Uppsala University)	Purification of 6xHis-tagged QnrB1 protein
pBAD-*mcbABCDEFG*	pBAD/His B	Gift of Dr. Mikhail Metelev (Uppsala University)	Encodes microcin B biosynthetic cluster (microcin B production)
pET21-GyrA	pET-21b(+)	this work (Dr. Jonathan Heddle)	Purification of untagged *E.coli* GyrA
pET21-GyrB	pET-21b(+)	this work (Dr. Jonathan Heddle)	Purification of untagged *E. coli* GyrB
pET21-3xFLAG-GyrB	pET-21b(+)	this work	Purification of FLAG-tagged GyrB
pET21-GyrA-FLAG	pET-21b(+)	this work	Purification of FLAG-tagged GyrA
pET28-GyrB47	pET-28a(+)	this work	Purification of 6xHis-GyrB47
pAJR10.18 (GyrB47)	pET-21a(+)	Gift of Anthony. Maxwell (John Innes Centre)	Purification of GyrB47 (untagged)
pAJ1 (GyrB43)	pET-21a(+)	Gift of Anthony. Maxwell (John Innes Centre)	Purification of GyrB43 (untagged)
pET28- HIS_FLAG_*qnrb1*	pET-28a(+)	this work	Purification of FLAG- and 6xHis-tagged QnrB1
pET28- HIS_FLAG_*albG*	pET-28a(+)	this work	Purification of FLAG- and 6xHis-tagged AlbG
pET28- HIS_*qnrb1ΔTTR*	pET-28a(+)	this work	Purification of QnrB1 loop deletion mutant
pEVOL-pBpF	pEVOL	Addgene #31190	Incorporation of *p*Bpa
pBAD-HIS_*qnrb1*_pBpa	pBAD/His B	this work	Incorporation of *p*Bpa
pBAD-HIS_*albG*_pBpa	pBAD/His B	this work	Incorporation of *p*Bpa

Table S3. List of primers used in the study

Primer Name	Primer sequence (5'-3')	Purpose
300f	CGGTATTCGGAATCTTGCAC	Amplification of pBR322 300 bp fragment
300r	GCGGTCCAATGATCGAAG	Amplification of pBR322 300 bp fragment
220f	CACTGGTCCCGCCACC	Amplification of pBR322 220 bp fragment
220r	CGATCCTTGAAGCTGTCC	Amplification of pBR322 220 bp fragment
147f	AGGCCATTATCGCCGGCATG	Amplification of pBR322 147bp fragment
147r	GCCTGGACAGCATGGCCTG	Amplification of pBR322 147bp fragment
133f	TATCGCCGGCATGGCGGC	Amplification of pBR322 133 bp fragment
133r	CAGCATGGCCTGCAACGC	Amplification of pBR322 133 bp fragment
100f	CGACGCGCTGGGCTACGTC	Amplification of pBR322 100 bp fragment
100r	CGCGGGCATCCCGATGCCG	Amplification of pBR322 100 bp fragment
76f	CTACGTCTTGCTGGCGTTCGCGACGCGAGGCTGGATGGCCTTCCCCA TTATGATTCTTCTCGCTTCCGGCGGCATC	Annealing of 76 bp pBR322 fragment
76r	GATGCCGCCGGAAGCGAGAAGAATCATAATGGGGAAGGCCATCCA GCCTCGCGTCGCGAACGCCAGCAAGACGTAG	Annealing of 76 bp pBR322 fragment
For_NcoI_McbG	ATCCCATGGGGATGGATATAATAGAAAAAGAATCACAAAACGA	Cloning of tagless *mcbG* gene into pBAD HIS B plasmid
Rev_XhoI_McbG	ATCTCTCGAGTCATCCCCCTACAACCACTC	Cloning of tagless *mcbG* gene into pBAD HIS B plasmid
For_NcoI_AlbG	ATCCCATGGGGATGCCGGCCAAGACCCTTGAAAGCAAGG	Cloning of tagless *albG* gene into pBAD HIS B plasmid
Rev_XhoI_Albg	ATCTCTCGAGTCAATCGGACAGCTCGATATCCAGGCT	Cloning of tagless *albG* gene into pBAD HIS B plasmid
For_ AlbG_Δ91–97	GTCAACTGGACCAGCGCACAAGCGGGGGCGCTGTCGTTCGAGCGCT G	Cloning of tagless *albG* loop deletion Δ91–97 gene into pBAD HIS B plasmid
Rev_AlbG_Δ91–97	CAGCGCTCGAACGACAGCGCCCCCGCTTGTGCGCTGGTCCAGTTGA C	Cloning of tagless *albG* loop deletion Δ91–97 gene into pBAD HIS B plasmid

For_NcoI_QnrB1	ATCCCATGGGGATGGCTCTGGCACTCGTTGGCGAAA	Cloning of tagless *qnrb1* gene into pBAD HIS B plasmid
Rev_XhoI_QnrB1	CCGCTCGAGTTAACCAATCACCGCGATGCCAAGTCGCTCCAT	Cloning of tagless *qnrb1* gene into pBAD HIS B plasmid
For_NdeI_3xFLAG-GyrB	AATACATATGGACTACAAAGACCATGACGGTGATTATAAAGATCATGACATCGATTACAAGGATGACGATGACAAGTCGAATTCTTATGACTCCTC	Cloning of N-terminally 3xFLAG *E .coli gyrB* gene
Rev_GyrB_XhoI	AATACTCGAGTTAAATATCGATATTCGCCGCTTTCAGG	Cloning of *E. coli gyrB* gene
For_NdeI_GyrA	TTATCATATGAGCGACCTTGCGAGAG	Cloning of *E. coli gyrA* gene
Rev_XhoI_GyrA_FLAG	TTAACTCGAGTTACTTGTCGTCATCGTCTTTGTAGTCACCGCTACCTTCTTCTTCTGGCTCGTCGTC	Cloning of C-terminally FLAG-tagged *E. coli gyrA* gene
For_NdeI_GyrB47	AATACATATGCGCCGTAAAGGTGCGC	Cloning of *E. coli* GyrB47 (TOPRIM) subdomain
For_NcoI_6xHis_FLAG_QnrB1	ATTACCATGGGCCATCATCATCATCATCATAGCGGCGATTATAAGGACGATGACGATAAGAGCGGCATGGCTCTGGCACTCGTTGGCGAAA	Cloning of N-terminally 6xHIS FLAG *qnrB1* gene
For_NcoI_6xHis_FLAG_AlbG	ATTACCATGGGCCATCATCATCATCATCATAGCGGCGATTATAAGGACGATGACGATAAGAGCGGCATGCCGGCCAAGACCCTTG	Cloning of N-terminally 6xHIS FLAG *albG* gene
For_AlbG_D109TAG	TGCATCCTCAACTAGAGCTTGTTCTAC	Introducing amber stop codon in *albG* gene for residue D109
Rev_AlbG_D109TAG	GTAGAACAAGCTCTAGTTGAGGATGCA	Introducing amber stop codon in *albG* gene for residue D109
For_Qnrb1_N27TAG	ACATTTTTTTAGTGTGATTTTTCA	Introducing amber stop codon in *qnrb1* for residue N27
Rev_Qnrb1_N27TAG	TGAAAAATCACACTAAAAAAATGT	Introducing amber stop codon in *qnrb1* for residue N27
For_Qnrb1_R48TAG	CAGTTCTATGATTAGGAAAGCCAGAAA	Introducing amber stop codon in *qnrb1* for residue R48
Rev_Qnrb1_R48TAG	TTTCTGGCTTTCCTAATCATAGAACTG	Introducing amber stop codon in *qnrb1* for residue R48
For_Qnrb1_Q51TAG	GATCGTGAAAGCTAGAAAGGGTGC	Introducing amber stop codon in *qnrb1* for residue Q51
Rev_Qnrb1_Q51TAG	GCACCCTTTCTAGCTTTCACGATC	Introducing amber stop codon in *qnrb1* for residue Q51
For_Qnrb1_I65TAG	CTGAAAGATGCCTAGTTTAAAAGC	Introducing amber stop codon in *qnrb1* for residue Q65
Rev_Qnrb1_I65TAG	GCTTTTAAACTAGGCATCTTTCAG	Introducing amber stop codon in *qnrb1* for residue Q65
For_Qnrb1_M73TAG	AGCTGTGATTTATCATAGGCGGATTTT	Introducing amber stop codon in *qnrb1* for residue Q73

Rev_Qnrb1_M73TAG	AAAATCCGCCTATGATAAATCACAGCT	Introducing amber stop codon in *qnrb1* for residue Q73
For_Qnrb1_R77TAG	GCGGATTTTTAGAATTCCAGTGCG	Introducing amber stop codon in *qnrb1* for residue Q77
Rev_Qnrb1_R77TAG	CGCACTGGAATTCTAAAAATCCGC	Introducing amber stop codon in *qnrb1* for residue R77
For_Qnrb1_N78TAG	GCGGATTTTCGCTAGTCCAGTGCGCTG	Introducing amber stop codon in *qnrb1* for residue N78
Rev_Qnrb1_N78TAG	CAGCGCACTGGACTAGCGAAAATCCGC	Introducing amber stop codon in *qnrb1* for residue N78
For_Qnrb1_G83TAG	AGTGCGCTGTAGATTGAAATT	Introducing amber stop codon in *qnrb1* for residue G83
Rev_Qnrb1_G83TAG	AATTTCAATCTACAGCGCACT	Introducing amber stop codon in *qnrb1* for residue G83
For_Qnrb1_E85TAG	CTGGGCATTTAGATTCGCCAC	Introducing amber stop codon in *qnrb1* for residue E85
Rev_Qnrb1_E85TAG	GTGGCGAATCTAAATGCCCAG	Introducing amber stop codon in *qnrb1* for residue E85
For_Qnrb1_R90TAG	TGAAATTCGCCACTGCTAGGCACAAGG	Introducing amber stop codon in *qnrb1* for residue R90
Rev_Qnrb1_R90TAG	CCTTGTGCCTAGCAGTGGCGAATTTCA	Introducing amber stop codon in *qnrb1* for residue R90
For_Qnrb1_D95TAG	ACAAGGCGCATAGTTCCGCGGC	Introducing amber stop codon in *qnrb1* for residue D95
Rev_Qnrb1_D95TAG	GCCGCGGAACTATGCGCCTTGT	Introducing amber stop codon in *qnrb1* for residue D95
For_Qnrb1_I105TAG	ATGAATATGTAGACCACGCGCACC	Introducing amber stop codon in *qnrb1* for residue I105
Rev_Qnrb1_I105TAG	GGTGCGCGTGGTCTACATATTCAT	Introducing amber stop codon in *qnrb1* for residue I105
For_Qnrb1_T106TAG	AATATGATCTAGACGCGCACCTGG	Introducing amber stop codon in *qnrb1* for residue T106
Rev_Qnrb1_T106TAG	CCAGGTGCGCGTCTAGATCATATT	Introducing amber stop codon in *qnrb1* for residue T106
For_Qnrb1_T107TAG	AATATGATCACCTAGCGCACCTGGTTT	Introducing amber stop codon in *qnrb1* for residue T107
Rev_Qnrb1_T107TAG	AAACCAGGTGCGCTAGGTGATCATATT	Introducing amber stop codon in *qnrb1* for residue T107

For_Qnrb1_R108TAG	ATGATCACCACGTAGACCTGGTTTTGT	Introducing amber stop codon in *qnrb1* for residue R108
Rev_Qnrb1_R108TAG	ACAAAACCAGGTCTACGTGGTGATCAT	Introducing amber stop codon in *qnrb1* for residue R108
For_Qnrb1_T109TAG	ATCACCACGCGCTAGTGGTTTTGTA	Introducing amber stop codon in *qnrb1* for residue T109
Rev_Qnrb1_T109TAG	TACAAAACCACTAGCGCGTGGTGAT	Introducing amber stop codon in *qnrb1* for residue T109
For_Qnrb1_F111TAG	CGCACCTGGTAGTGTAGCGCATAT	Introducing amber stop codon in *qnrb1* for residue F111
Rev_Qnrb1_F111TAG	ATATGCGCTACACTACCAGGTGCG	Introducing amber stop codon in *qnrb1* for residue F111
For_Qnrb1_S113TAG	TGGTTTTGTTAGGCATATATCACG	Introducing amber stop codon in *qnrb1* for residue S113
Rev_Qnrb1_S113TAG	CGTGATATATGCCTAACAAAACCA	Introducing amber stop codon in *qnrb1* for residue S113
For_Qnrb1_T117TAG	AGCGCATATATCTAGAATACCAATCTA	Introducing amber stop codon in *qnrb1* for residue T117
Rev_Qnrb1_T117TAG	TAGATTGGTATTCTAGATATATGCGCT	Introducing amber stop codon in *qnrb1* for residue T117
For_Qnrb1_N120TAG	ACGAATACCTAGCTAAGCTACGCC	Introducing amber stop codon in *qnrb1* for residue N120
Rev_Qnrb1_N120TAG	GGCGTAGCTTAGCTAGGTATTCGT	Introducing amber stop codon in *qnrb1* for residue N120
For_Qnrb1_Y123TAG	ACCAATCTAAGCTAGGCCAATTTTTCG	Introducing amber stop codon in *qnrb1* for residue Y123
Rev_Qnrb1_Y123TAG	CGAAAAATTGGCCTAGCTTAGATTGGT	Introducing amber stop codon in *qnrb1* for residue Y123
For_Qnrb1_N125TAG	AGCTACGCCTAGTTTTCGAAAGTC	Introducing amber stop codon in *qnrb1* for residue N125
Rev_Qnrb1_N125TAG	GACTTTCGAAAACTAGGCGTAGCT	Introducing amber stop codon in *qnrb1* for residue N125
For_Qnrb1_E132TAG	GTCGTGTTGTAGAAGTGTGAGCTG	Introducing amber stop codon in *qnrb1* for residue E132
Rev_Qnrb1_E132TAG	CAGCTCACACTTCTACAACACGAC	Introducing amber stop codon in *qnrb1* for residue E132
For_Qnrb1_E138TAG	TGTGAGCTGTGGTAGAACCGTTGG	Introducing amber stop codon in *qnrb1* for residue E138

Rev_Qnrb1_E138TAG	CCAACGGTTCTACCACAGCTCACA	Introducing amber stop codon in *qnrb1* for residue E138
For_Qnrb1_R140TAG	CTGTGGGAAAACTAGTGGATAGGT	Introducing amber stop codon in *qnrb1* for residue R140
Rev_Qnrb1_R140TAG	ACCTATCCACTAGTTTTCCCACAG	Introducing amber stop codon in *qnrb1* for residue R140
For_Qnrb1_L147TAG	GGTGCCCAGGTATAGGGCGCGACGTTC	Introducing amber stop codon in *qnrb1* for residue L147
Rev_Qnrb1_L147TAG	GAACGTCGCGCCCTATACCTGGGCACC	Introducing amber stop codon in *qnrb1* for residue L147
For_Qnrb1_D155TAG	TTCAGTGGTTCATAGCTCTCC	Introducing amber stop codon in *qnrb1* for residue D155
Rev_Qnrb1_D155TAG	GGAGAGCTATGAACCACTGAA	Introducing amber stop codon in *qnrb1* for residue D155
For_Qnrb1_S157TAG	TTCAGATCTCTAGGGCGGCGA	Introducing amber stop codon in *qnrb1* for residue S157
Rev_Qnrb1_S157TAG	TCGCCGCCCTAGAGATCTGAA	Introducing amber stop codon in *qnrb1* for residue S157
For_Qnrb1_R167TAG	ACTTTCGACTGGTAGGCAGCAAAC	Introducing amber stop codon in *qnrb1* for residue R167
Rev_Qnrb1_R167TAG	GTTTGCTGCCTACCAGTCGAAAGT	Introducing amber stop codon in *qnrb1* for residue R167
For_Qnrb1_D175TAG	ACACATTGCTAGCTGACCAATTCG	Introducing amber stop codon in *qnrb1* for residue D175
Rev_Qnrb1_D175TAG	CGAATTGGTCAGCTAGCAATGTGT	Introducing amber stop codon in *qnrb1* for residue D175
For_Qnrb1_D185TAG	TTGGGTGACTTATAGATTCGGGGC	Introducing amber stop codon in *qnrb1* for residue D185
Rev_Qnrb1_D185TAG	GCCCCGAATCTATAAGTCACCCAA	Introducing amber stop codon in *qnrb1* for residue D185
For_Qnrb1_R187TAG	GACTTAGATATTTAGGGCGTTGAT	Introducing amber stop codon in *qnrb1* for residue R187
Rev_Qnrb1_R187TAG	ATCAACGCCCTAAATATCTAAGTC	Introducing amber stop codon in *qnrb1* for residue R187

REFERENCES

1. Jacoby,G.A., Corcoran,M.A., Mills,D.M., Griffin,C.M. and Hooper,D.C. (2013) Mutational analysis of quinolone resistance protein QnrB1. *Antimicrob. Agents Chemother.*, **57**,

5733–5736.
